# Experimental study on shear mechanical properties of concrete joints under different unloading stress paths

**DOI:** 10.1371/journal.pone.0310694

**Published:** 2024-09-19

**Authors:** Zhezhe Zhang, Baohua Guo, Shengjin Cheng, Pengbo Zhong, Chuangwei Zhu

**Affiliations:** 1 School of Energy Science and Engineering, Henan Polytechnic University, Jiaozuo, China; 2 Collaborative Innovation Center of Coal Work Safety, Jiaozuo, China; 3 Jiaozuo Engineering Research Center of Road Traffic and Transportation, Henan Polytechnic University, Jiaozuo, Henan, China; University of Vigo, SPAIN

## Abstract

In order to study the shear mechanical properties of rock joint under different unloading stress paths, the RDS-200 rock joint shear test system was used to carry out direct shear tests on concrete joint specimens with five different morphologies under the CNL path and different unloading stress paths. The unloading stress paths include unloading normal load and maintaining constant shear load (UNLCSL), unloading normal load and unloading shear load (UNLUSL), unloading normal load and increasing shear load (UNLISL). The results show that the peak shear strength, cohesion, internal friction angle, pre-peak shear stiffness and residual shear strength of concrete joints under CNL path increases with the increasing *JRC* and normal stress. Under the UNLCSL path, under the same initial shear stress τ_1_, instability normal stress σ_*i*_ decreases with the increasing *JRC*, and normal stress unloading amount Δσ_n_ increases with the increasing *JRC*. Under the same *JRC*, σ_*i*_ increases with the increase of τ_1_, and Δσ_n_ decreases with the increasing τ_1_. Under the same *JRC* and σ_*i*_, τ_*i*_ is significantly smaller under the UNLCSL path than the CNL path. Under the same *JRC*, the cohesion under the UNLCSL path is less than the CNL path, and the internal friction angle is higher than that the CNL path. Under the same *JRC* and σ_*i*_, τ_*i*_ is the largest under the path of CNL and UNLISL, followed by the UNLCSL path, and τ_*i*_ under the UNLUSL path is the smallest. Compared with the CNL path, the variation range of the specimen internal friction angle is within 3% while the average decrease percentage of the specimen cohesion reaches 37.6% under the UNLCSL path, UNLISL, and UNLUSL. Therefore, it can be inferred that the decrease in cohesion caused by normal unloading is the main reason for the decrease in joint instability shear strength. After introducing the correction coefficient *k* of cohesion to modify the Mohr-Coulomb criterion, the maximum average relative error after correction is only 3.5%, which is significantly improved compared with the maximum average relative error of 56.9% before correction. The research conclusions can provide some reference for the accurate estimation of shear bearing capacity of rock joints under different unloading stress paths, which is of great significance to the stability evaluation and disaster prevention of rock mass engineering.

## 1. Introduction

The engineering rock mass contains a large number of joints of different scales. Joints are widely distributed in rock mass engineering such as caverns, tunnels and slopes. The existence of joints affects the integrity of rock mass and reduces the strength of rock mass. The excavation disturbance of mining engineering, slope engineering and underground space is a typical unloading process. Due to excavation unloading, it is bound to cause unloading deformation in the adjacent surface area, which is easy to induce shear slip dynamic instability failure [1]. Therefore, it is of great engineering and theoretical significance to study shear mechanical behavior of joint under normal unloading conditions. In recent years, as one of the hot research directions in the field of geotechnical engineering, unloading rock mass mechanics has attracted the attention of many scholars. The deformation caused by unloading will directly affect the stability and safety of rock mass engineering [2–5].

The scholars studied the shear characteristics of rock joints based on the condition of constant normal stress, such as based on a series of conventional direct shear test results, Grasselli et al. [6] studied the influence of the morphology of the structural plane on its shear strength in detail, and on this basis, a failure criterion of rock mass shear strength considering the morphology of the structural plane was proposed. Asadollahi et al. [7] statistically analyzed 372 sets of joint direct shear test results under normal stress conditions, constructed the corresponding relationship between peak dilatancy angle and wall rock strength, and modified the *JRC*-*JCS* peak shear strength calculation model proposed by Barton. H. J. Schneider [8] carried out direct shear tests under normal stress conditions and studied the relationship between peak dilatancy angle and normal stress. K. A. Hossaini et al. [9] studied the influence of roughness change and joint deformation on the shear mechanical behavior of rock joints, and established a peak shear strength model considering the influence of joint convex deformation characteristics. Based on the direct shear test results under constant normal stress conditions, JING [10], LEE et al. [11] proposed the prediction model of joint peak shear strength under constant normal stress conditions. M.R. Shen and Q.Z. Zhang [12] used cement mortar to cast specimens with regular serrated joints, and conducted direct shear tests under normal stress conditions of 2–5 MPa to study the shear characteristics of regular serrated joints. P. Cao et al. [13] used cement mortar specimens with different joint morphologies to study the specific effects of morphology on joint shear characteristics. In addition. X.B. Zhang et al. [14] conducted direct shear tests on joint specimens made of concrete materials, and studied the effects of shear direction, specimen size and effective normal stress on joint shear characteristics. Through the direct shear test results, J. Zhao [15–17] found that the coincidence degree will affect the prediction accuracy of the *JRC*-*JCS* model, so the joint coincidence coefficient *JMC* is introduced, and the *JRC*-*JMC* shear strength criterion is proposed. Based on the Grasselli model, C.C. Xia, and Z.C. Tang et al. [18–20] studied the relationship between the peak dilatancy angle of the joint and the morphological parameters. Considering the dilatancy effect, a new peak shear strength formula of the joint was obtained, which conforms to the form of Mohr-Coulomb law. In the subsequent experimental study, they also considered the relationship between dilatancy angle and normal stress, proposed an empirical dilatancy angle model in hyperbolic form, and then established a new joint peak shear strength formula. J. Yang et al. [21] conducted direct shear tests on granite and red sandstone rock joint specimens obtained by artificial splitting under normal stress conditions. A joint peak shear strength model was successfully established based on the maximum effective shear dip angle and roughness parameters in the three-dimensional morphology parameters. T. Cheng et al. [22, 23] carried out the direct shear test of rock joints under the condition of constant normal stress on the irregular sandstone and granite joint specimens prepared by the splitting method. The empirical formula of shear strength of irregular rock joints considering roughness and the constitutive prediction model of shear stress-displacement curves of rock joints with different roughness is proposed. L.L. Jin, R.C. Liu, H. Shen et al. [24–26] have studied the shear strength model of rock joints under constant normal stress.

The surface morphology of the joint will directly affect the strength and deformation characteristics of the rock mass structure. Many scholars have studied the influence of joint morphology on joint shear characteristics [27–29]. Roughness and undulation are usually used to measure the morphology of irregular and regular toothed joints. Barton [30] first proposed the concept of joint roughness coefficient *JRC*, and based on more than 200 sets of direct shear test results, the joint roughness coefficient *JRC* is divided into 10 levels according to the degree of roughness. Asadollahi [31], C.C. Xia et al. [32] studied the effect of roughness on peak shear displacement under normal stress. Z.Q. Zhang et al. [33] used the ten standard joint curves proposed by Barton, and used PFC software to establish the rock joint specimen model. The relationship between the roughness coefficient *JRC* and the peak shear strength and the crack propagation law of the specimen under different *JRC* and different normal stress conditions were studied. B.C. Wu et al. [34] carried out direct shear tests on joint specimens with different roughness under normal stress conditions, and obtained that the peak shear strength of joints increased with the increase of roughness. R.H. Cao et al. [35] carried out numerical simulation shear tests on joint models with five roughness coefficients under normal stress conditions. Based on the numerical simulation results, the influence of roughness on joint failure characteristics was analyzed microscopically. Abolfazli et al. [36] analyzed the correlation between *JRC* and various parameters used for roughness evaluation by conducting direct shear tests on rock joints with different roughness coefficients under normal stress conditions. Some scholars have studied the influence of roughness on the shear mechanical properties of the two-body interface [28, 37–40].

In recent years, a few scholars have carried out research on joint shear characteristics under normal unloading conditions. D. Huang et al. [41] conducted a shear test on red sandstone with joint cracks under normal unloading conditions. It was found that normal unloading not only reduced the shear capacity but also caused a significant increase in deformation in the unloading direction. C. Yang et al. [42] conducted a shear test on non-interpenetrated jointed cement mortar specimens under normal unloading conditions, and obtained that the shear strength obtained under normal unloading conditions in the range of normal stress is lower than that obtained under constant normal stress. After Zhu et al. [43] tested the intact red sandstone, he pointed out that the cohesion of the specimen under normal unloading conditions decreased significantly with the increase of τ_1_, and the internal friction angle increased slightly, and a large number of fragments were peeled off near the main cracks after the failure of the specimen, while the cracks in the conventional direct shear test were relatively flat. There are obvious differences in the shear mechanical properties and deformation characteristics of joints under normal unloading conditions and constant normal stress conditions. Therefore, it is necessary to study the shear characteristics of joints under normal unloading conditions.

From the current research results, many scholars in China and other countries in the world mainly focus on the study of joint shear characteristics under the CNL path. Although a small number of scholars have carried out the study of joint shear characteristics under normal unloading stress path, most of them use complete specimens or specimens with prefabricated cracks, mainly considering the influence of factors such as the number of cracks, geometric distribution, crack angle and load level. However, there are relatively few studies on the shear characteristics of persistent joints under normal unloading stress path. Only by fully grasping the shear characteristics of joint fracture during unloading, can we put forward reasonable measures for slope treatment, roadway, and chamber maintenance. Based on this, the RDS-200 rock joint shear test system was used to carry out direct shear tests on concrete joint specimens with five different morphologies under CNL and different unloading stress paths, and the shear mechanical properties of different *JRC* concrete joints under different unloading stress paths were studied.

## 2. Experimental testing

### 2.1 Preparation process of concrete joint specimens

**Fig 1:**
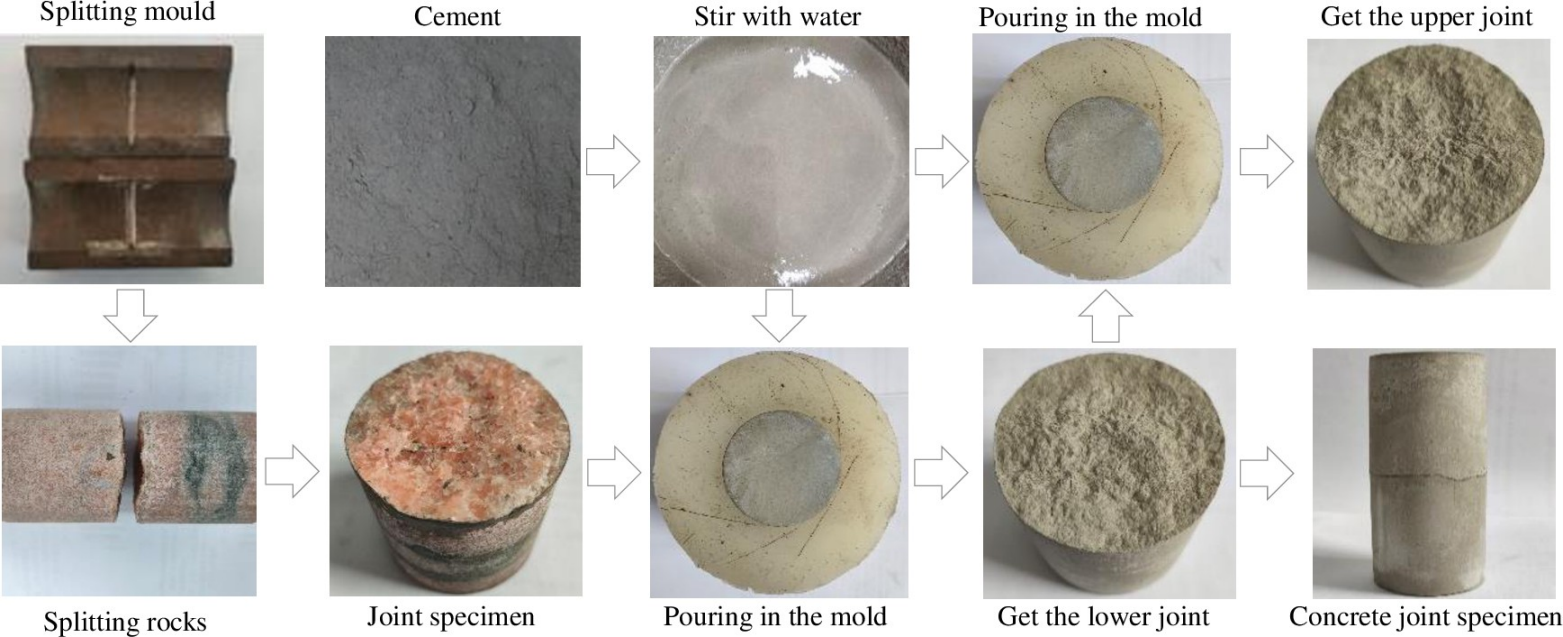
Preparation process of concrete joint specimens.

**Fig 2:**
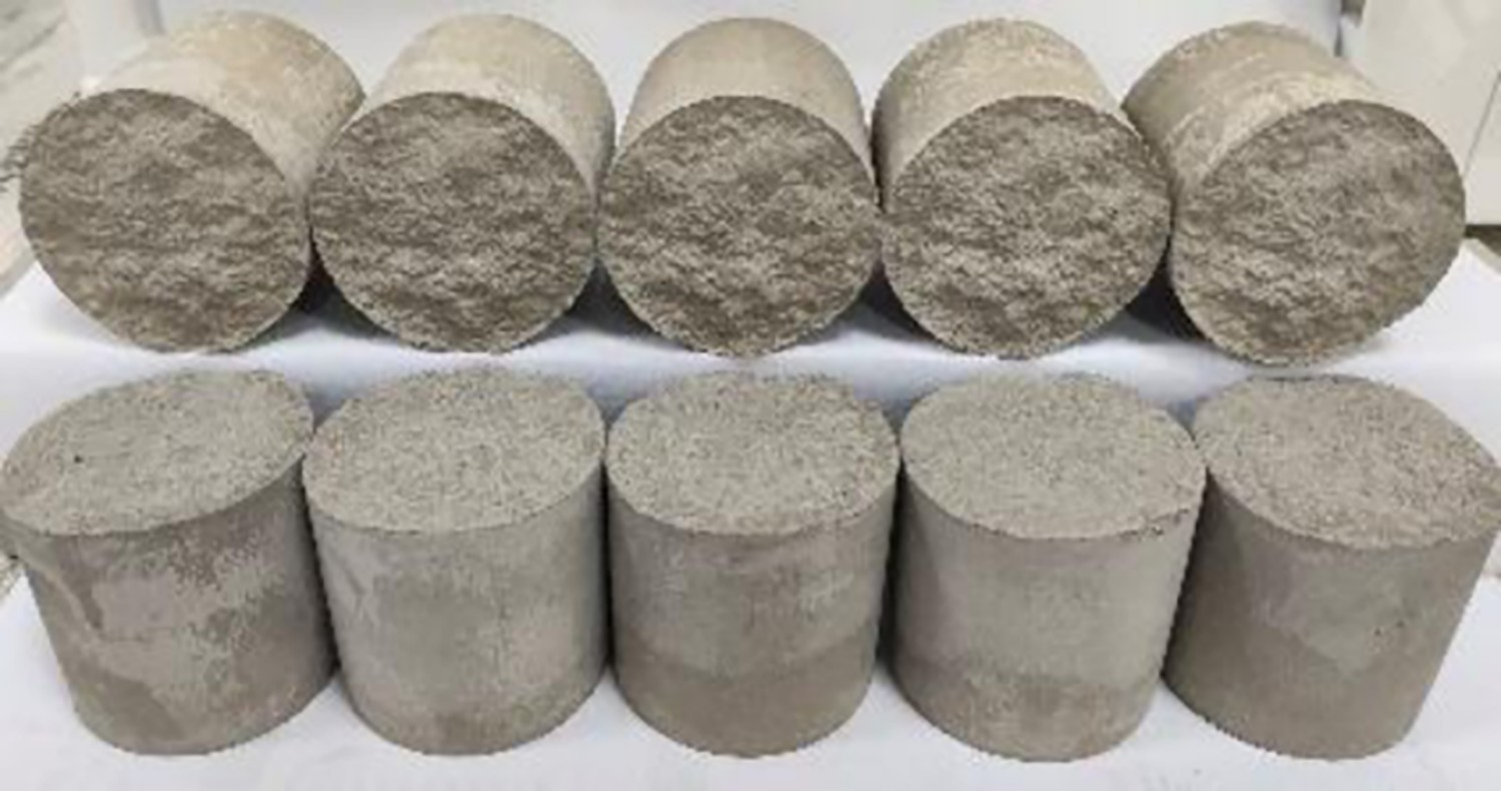
Concrete joint specimens with the same morphology.

The morphology of rock joints is one of the important factors affecting the mechanical properties of rock joints. Since the direct shear test of rock joints is a destructive test, it is impossible to carry out shear test research on the same natural joint under different conditions. To reduce the influence of joint morphology on the test results, this paper replicates five different morphologies of rock joint to obtain concrete joint specimens with the same joint morphology. After splitting the five standard cylindrical granite specimens near the axial midpoint by using the splitting mould, a relatively complete half was selected as the joint to be replicated, and the joint of one of the specimens were polished. The specimens were selected from the high-strength sulphochlorate quick-setting cement produced by Shandong Bokai New Material Technology Co., Ltd., which was fully stirred according to the water-cement ratio of 0.25, and then poured on the split rock joint with the help of silica gel mould to form a concrete joint specimen. After pouring, it was placed for about 15 minutes to be demoulded after the cement solidified. The process of obtaining concrete joint specimens by pouring concrete joints again to obtain complete concrete joint specimens with the same joint morphology is shown in Fig 1. To ensure the complete separation of the poured concrete from the original joint, the diluted concrete release agent is uniformly applied to the original joint surface before pouring. After many tests, the dilution ratio of the concrete release agent to water is finally determined to be 1:5, which can not only ensure the complete separation of the concrete from the original joint, but also ensure the minimum impact on the strength of the joint surface. The prepared concrete joint specimens are shown in Fig 2. After 28 days of curing under standard curing conditions, the direct shear test was carried out to study the shear characteristics of concrete joints under the CNL path and different unloading stress paths. The basic mechanical parameters of the standard specimens after curing are shown in Table 1. In Table 1, σ_c_ is uniaxial compressive strength, *E* is elastic modulus, *R*_t_ is tensile strength, *φ* is internal friction angle, *c* is cohesion, *μ* is Poisson’s ratio.

**Table 1:** Basic mechanical parameters of concrete specimens.

Specimen type	*σ*_c_(MPa)	*E*(GPa)	*R*_t_(MPa)	*φ*(°)	*c*(MPa)	*μ*
Concrete	65.69	8.28	1.74	36.8	14.89	0.1

### 2.2 Acquisition of joint morphology parameters

**Fig 3:**
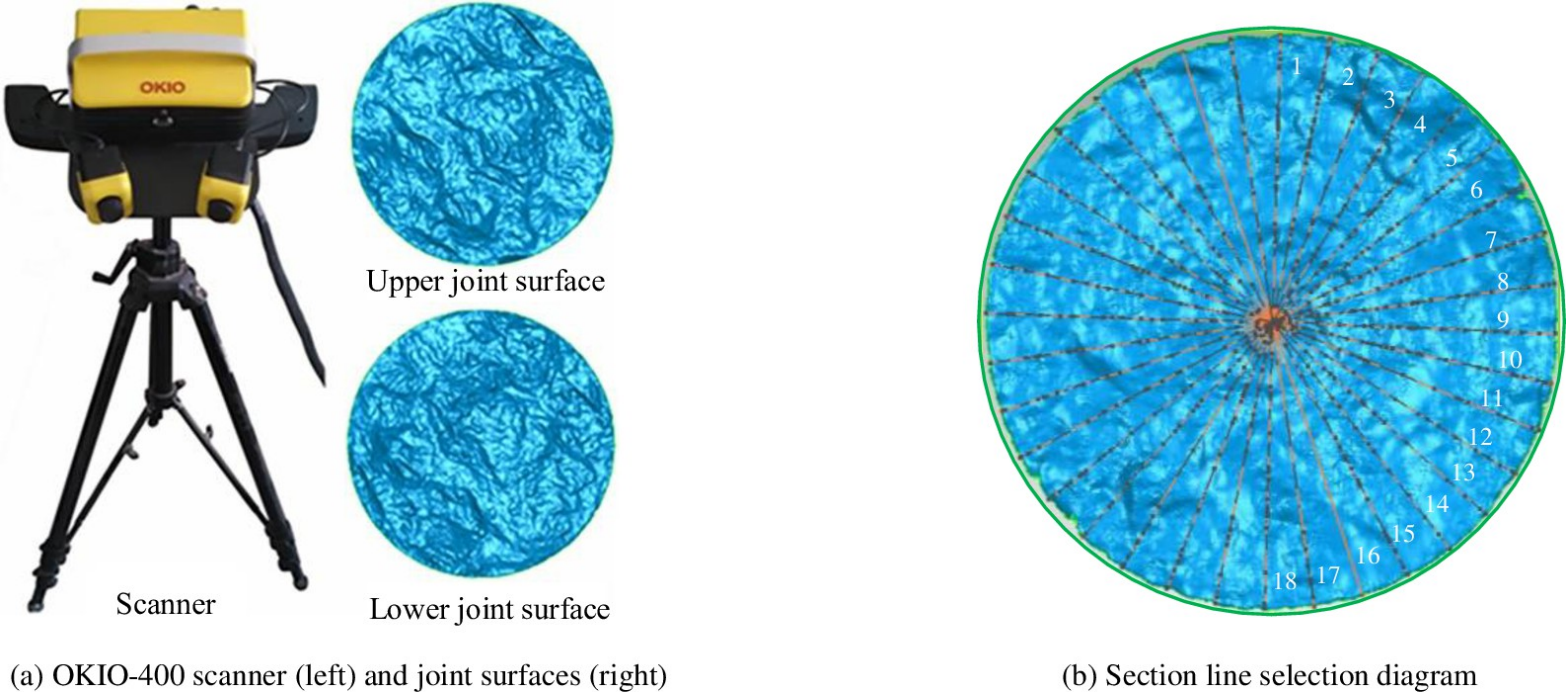
OKIO 3D scanner, 3D views of the fracture surfaces and selection of section line. (a) OKIO-400 scanner (left) and joint surfaces (right). (b) Section line selection diagram.

**Fig 4:**
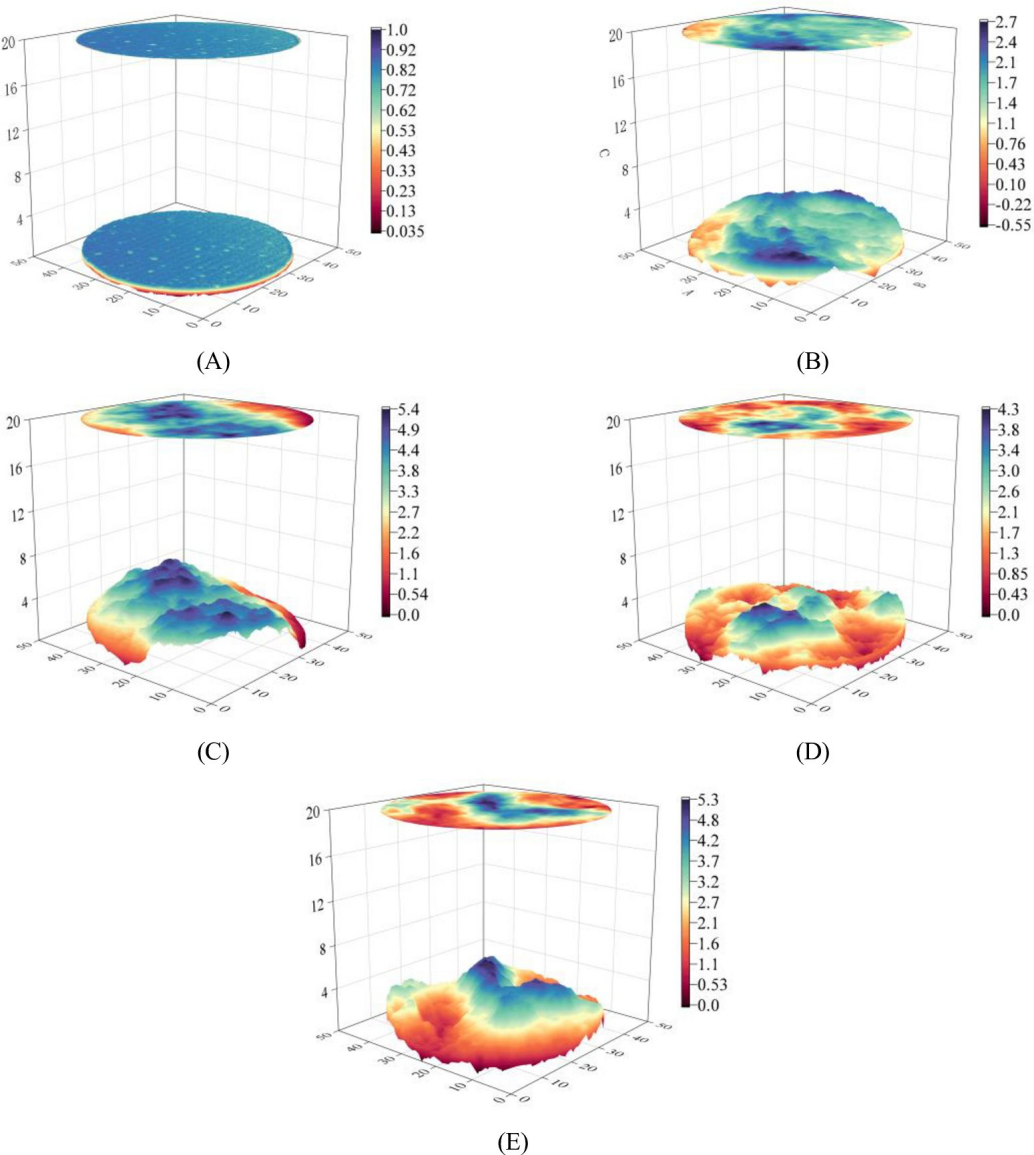
Three-dimensional topography of concrete joints.

After the completion of the five groups of concrete joints, the joint surface was scanned by Tianyuan OKIO-400 three-dimensional topography scanner [44]. The scanner adopted a non-contact surface scanning method, automatic splicing method of marker points, and control accuracy of the global error control module. The maximum measurement range is 400 mm×300 mm, and the measurement accuracy can reach 0.03~0.02 mm. The Tianyuan OKIO-400 three-dimensional scanner is used to scan the rock joint surface to obtain the joint surface morphology and extract the three-dimensional coordinate data of the profile line. The joint roughness parameters are obtained by the supporting rock joint morphology processing system software. The scanner and the joint surface scanning results are shown in Fig 3(A). The three-dimensional morphology of the joint surface obtained by treatment is shown in Fig 4.

To further calculate the roughness coefficient of concrete joints, 18 profile lines are selected with the center of the joint surface as the axis (see Fig 3(B)). By exporting the point cloud data, the slope root means square Z_*2j*_ [45] of the jth profile line can be calculated. The calculation formula of Z_*2j*_ is given as follows:

$$Z_{2j}={\left[\frac{1}{(m-1)\Delta x^{2}}\sum_{i=1}^{m-1}{\left(Z_{i+1}-Z_{i}\right)}^{2}\right]}^{\frac{1}{2}}$$ (1)

where Z_*i*_ is the height coordinate of the ith sampling point on the section line(mm); *i* is the serial number of sampling point, natural number; *m* is the total number of sampling points; Δ*x* is the sampling interval(mm). Substitute the Z_*2j*_ of each profile line into the following formula [45, 46], and the joint roughness coefficient *JRC*_*j*_ of each profile line is calculated.

$$JRC_{j}=32.69+32.98\mathrm{lg}Z_{2j}$$ (2)

where *JRC*_*j*_ is the roughness coefficient of the profile of the *j*th section. The average value of *JRC*_*j*_ of all the profile lines of the joint surface is *JRC*, which is the joint roughness coefficient that can reflect the three-dimensional morphology of the joint surface [23]. The *JRC* value of each group of specimens are calculated as shown in Table 2.

**Table 2:** *JRC* values of concrete joint specimens of each group.

Specimen group	A	B	C	D	E
*JRC*	0	8.01	11.06	12.26	16.37

### 2.3 Experimental facilities

**Fig 5:**
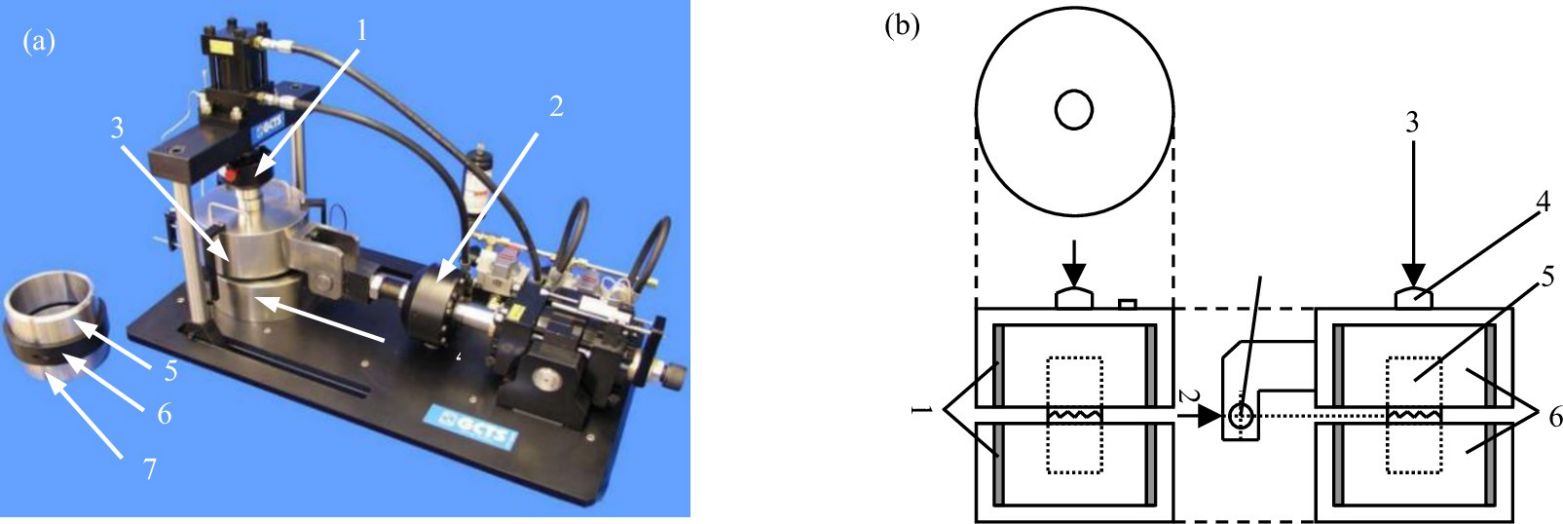
RDS-200 direct shear system and the three-view diagram of shear box (a) RDS-200 rock direct shear system, 1-normal actuator; 2-shearing actuator; 3-upper shear box; 4-lower shear box; 5-upper shear ring; 6-gasket; 7-lower shear ring (b) three views of shear box, 1-shear ring; 2-shear load; 3-normal load; 4-pressure head; 5-test specimen; 6-shear box.

The shear test of concrete joints was carried out by RDS-200 rock direct shear apparatus [22] developed by GCTS company in the United States, as shown in Fig 5. The equipment is controlled by the electro-hydraulic servo control system, which can carry out the shear test under CNL and CNS conditions. The maximum load of the shear actuator and normal actuator is 10t and 5t respectively, and the accuracy is 0.01kN. The maximum shear and normal strokes are 25mm and 24mm, respectively, and the accuracy is 0.001 mm.

### 2.4 Test method and test scheme

**Fig 6:**
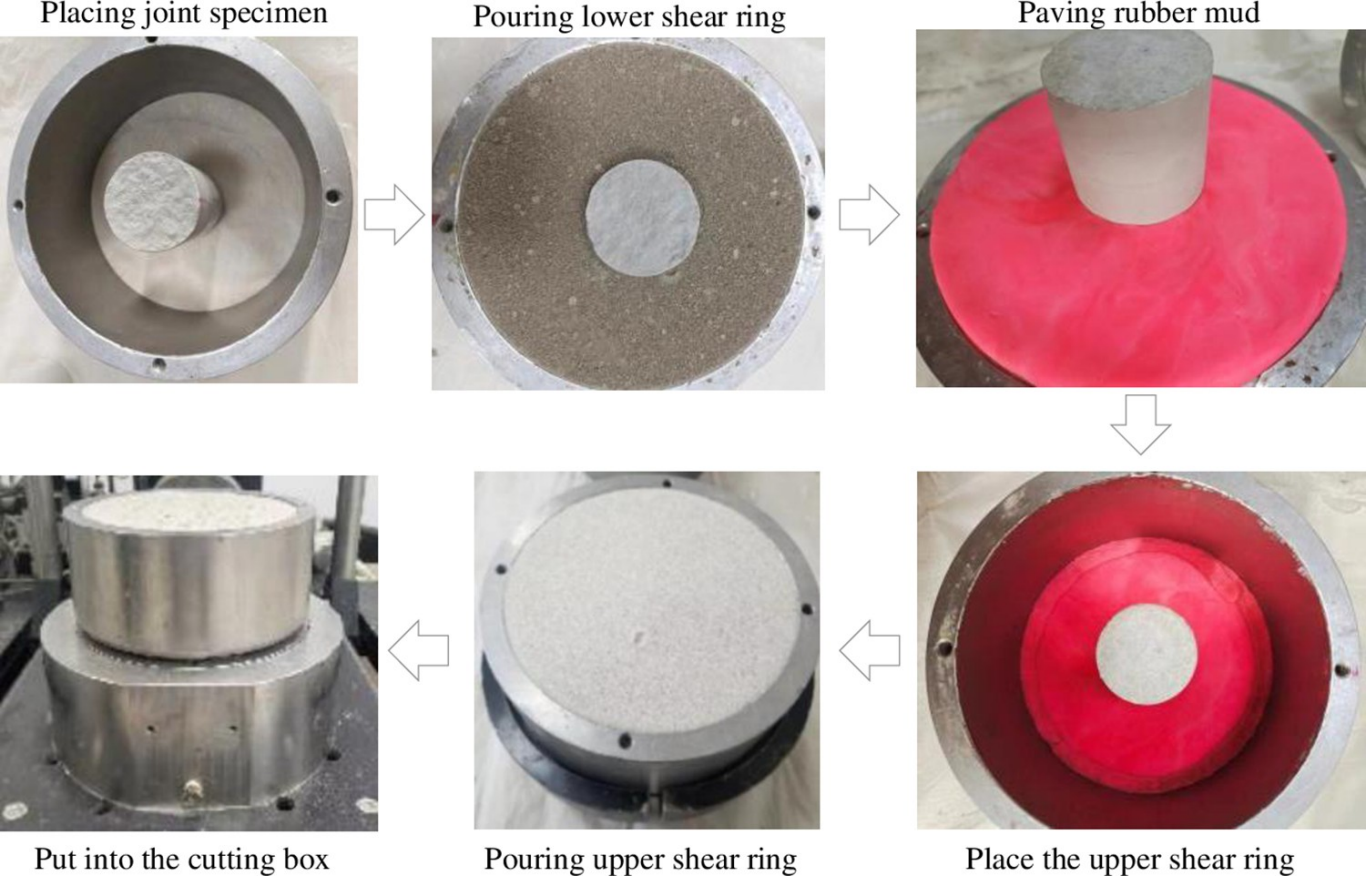
Specimen packaging process.

Before the shear test, the specimen needs to be packaged and put into the shear box for the direct shear test. The specific packaging process is shown in Fig 6. It should be noted that to reduce the influence of anisotropy, the shear direction of the joint surface should be unified and marked on the shear ring before packaging.

According to the different control methods and unloading stress paths, the four groups of test schemes were designed for five kinds of *JRC* concrete joints, which were group I: CNL, group Ⅱ: unloading normal load and maintaining constant shear load (referred to as UNLCSL), group Ⅲ: unloading normal load and increase shear load (referred to as UNLISL), group Ⅳ: unloading normal load and unloading shear load (referred to as UNLUSL). In addition to the group IV test, the displacement control method adopts the loading and unloading rate of 1 mm/min, and the stress control method adopts the loading and unloading rate of 2 MPa/min, as follows:

Group Ⅰ: CNL. The first step is to apply normal stress to the set value of 2, 4, 6, 8, 10 MPa by stress control. The second step: keep the normal stress unchanged, use the displacement control method to carry out shear loading, and end the test when the shear displacement is 8 mm. Five kinds of concrete joint specimens with different *JRC* were tested, and a total of 25 groups of tests. The specimens were named ⅠA-2~ⅠA-10, ⅠB-2~ⅠB-10, ⅠC-2~ⅠC-10, ⅠD-2~ⅠD-10 and ⅠE-2~ⅠE-10.

Group Ⅱ: UNLCSL. The first step is to apply normal stress to 8 MPa by stress control. In the second step, keeping the normal stress unchanged, the shear stress is applied to 0.3*τ*_p_,0.4*τ*_p_,0.5*τ*_p_,0.6*τ*_p_,0.7*τ*_p_ by stress control method (*τ*_p_ represents the peak shear strength of each *JRC* concrete joint under normal stress of 8 MPa); the third step: keep the shear stress unchanged, the normal unloading until the specimen is destroyed. Five kinds of concrete joint specimens with different *JRC* were tested, and a total of 25 groups of tests. The specimens were named ⅡA-0.3~ⅡA-0.7, ⅡB-0.3~ⅡB-0.7, ⅡC-0.3~ⅡC-0.7, ⅡD-0.3~ⅡD-0.7 and ⅡE-0.3~ⅡE-0.7.

Group Ⅲ: UNLISL. The first and second steps were consistent with the group II test. The third step: the stress control method is used to perform shear loading at a loading rate of 2MPa/min, and the stress control method is used to perform normal unloading at an unloading rate of 2MPa/min until the specimen is destroyed. Five groups of concrete joint specimens with a *JRC* of 11.06 were tested, and the specimens were named IIIC-0.3~IIIC-0.7.

Group Ⅳ: UNLUSL. The first and second steps were consistent with the group II test. The third step: the shear unloading is carried out by the stress control mode at the loading rate of 0.5MPa/min, and the normal unloading is carried out by the stress control mode at the unloading rate of 2MPa/min until the specimen is destroyed. Five groups of concrete joint specimens with a *JRC* of 11.06 were tested, and the specimens were named IVC-0.3~IVC-0.7.

**Fig 7:**
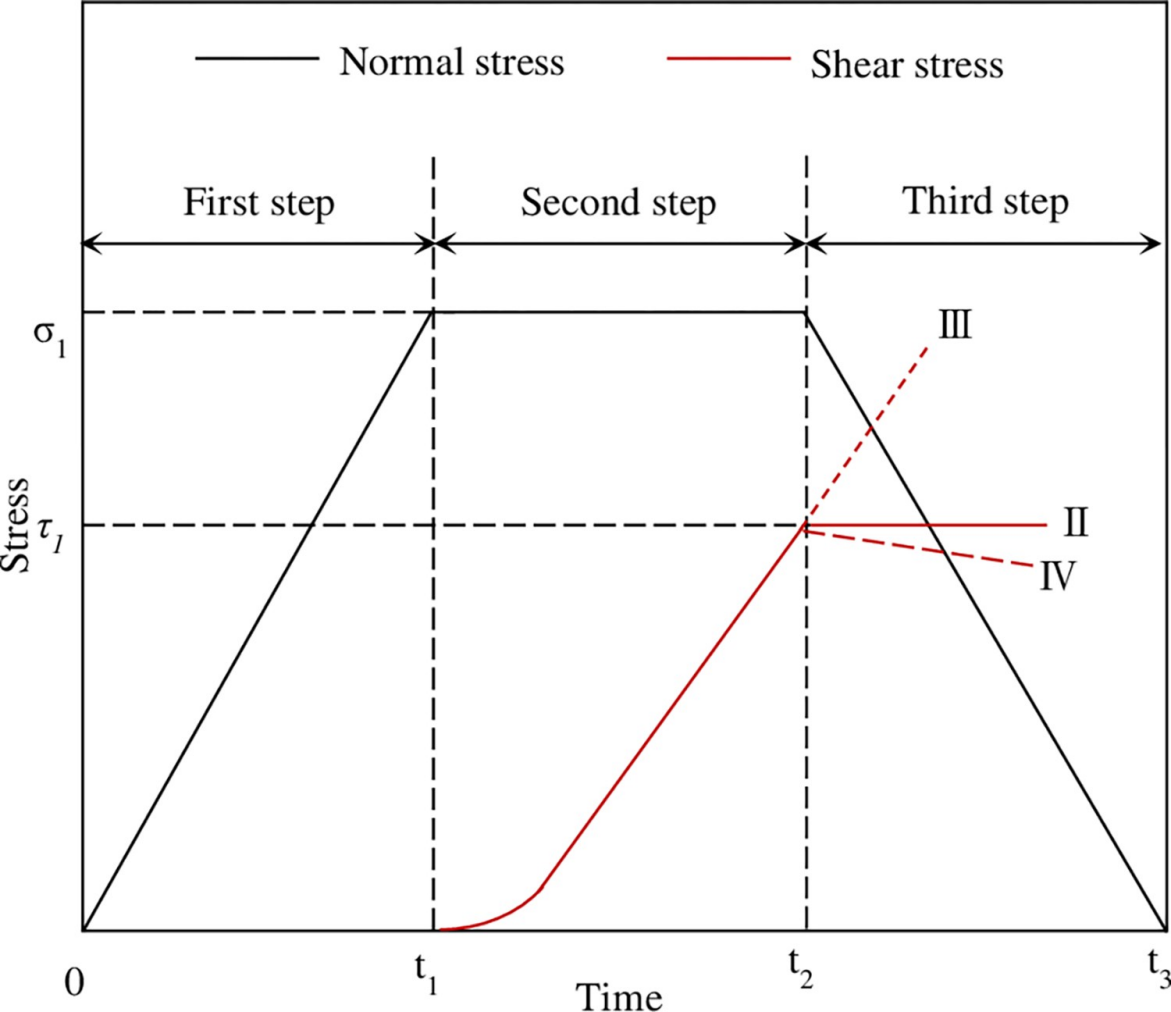
Stress path diagram of Ⅱ, Ⅲ and Ⅳ groups of specimens.

To intuitively show the difference between the stress paths of the three groups II, III and IV, the schematic diagram of the stress paths of the three groups II, III and IV is shown in Fig 7. In Fig 7, σ_1_ is the initial normal stress, which represents the normal stress at the beginning of normal unloading, and τ_1_ is the initial shear stress, which refers to the shear stress at the beginning of normal unloading. The essence of the stress path of the four groups of specimens is to reduce the difference between the normal stress σ_n_ and the shear stress τ (σ_n_-τ) to cause the shear instability of the concrete joints. The difference between the normal stress σ_n_ and the shear stress τ of the group III test is the largest, while the difference between the normal stress σ_n_ and the shear stress τ of the group IV test is the smallest. The specific test scheme is shown in Table 3.

**Table 3:** Test scheme.

Test group	Stress path	Specimen number	*JRC*	Normal stress (MPa)	σ_1_(MPa)	τ_1_(MPa)
Ⅰ	CNL	ⅠA-2~ⅠA-10	0	2, 4, 6, 8, 10	/	/
		ⅠB-2~ⅠB-10	8.01			
		ⅠC-2~ⅠC-10	11.06			
		ⅠD-2~ⅠD-10	12.26			
		ⅠE-2~ⅠE-10	16.37			
Ⅱ	UNLCSL	ⅡA-0.3~ⅡA-0.7	0	/	8	0.3τ_p_, 0.4τ_p_, 0.5τ_p_, 0.6τ_p_, 0.7τ_p_
		ⅡB-0.3~ⅡB-0.7	8.01			
		ⅡC-0.3~ⅡC-0.7	11.06			
		ⅡD-0.3~ⅡD-0.7	12.26			
		ⅡE-0.3~ⅡE-0.7	16.37			
Ⅲ	UNLISL	IIIC-0.3~IIIC-0.7	11.06	/	8	0.3τ_p_, 0.4τ_p_, 0.5τ_p_, 0.6τ_p_, 0.7τ_p_
Ⅳ	UNLUSL	IVC-0.3~IVC-0.7	11.06	/	8	0.3τ_p_, 0.4τ_p_, 0.5τ_p_, 0.6τ_p_, 0.7τ_p_

## 3. Experiment results and analysis

### 3.1 Shear test results and analysis under the CNL path

#### 3.1.1 Shear stress-shear displacement curve characteristics

**Fig 8:**
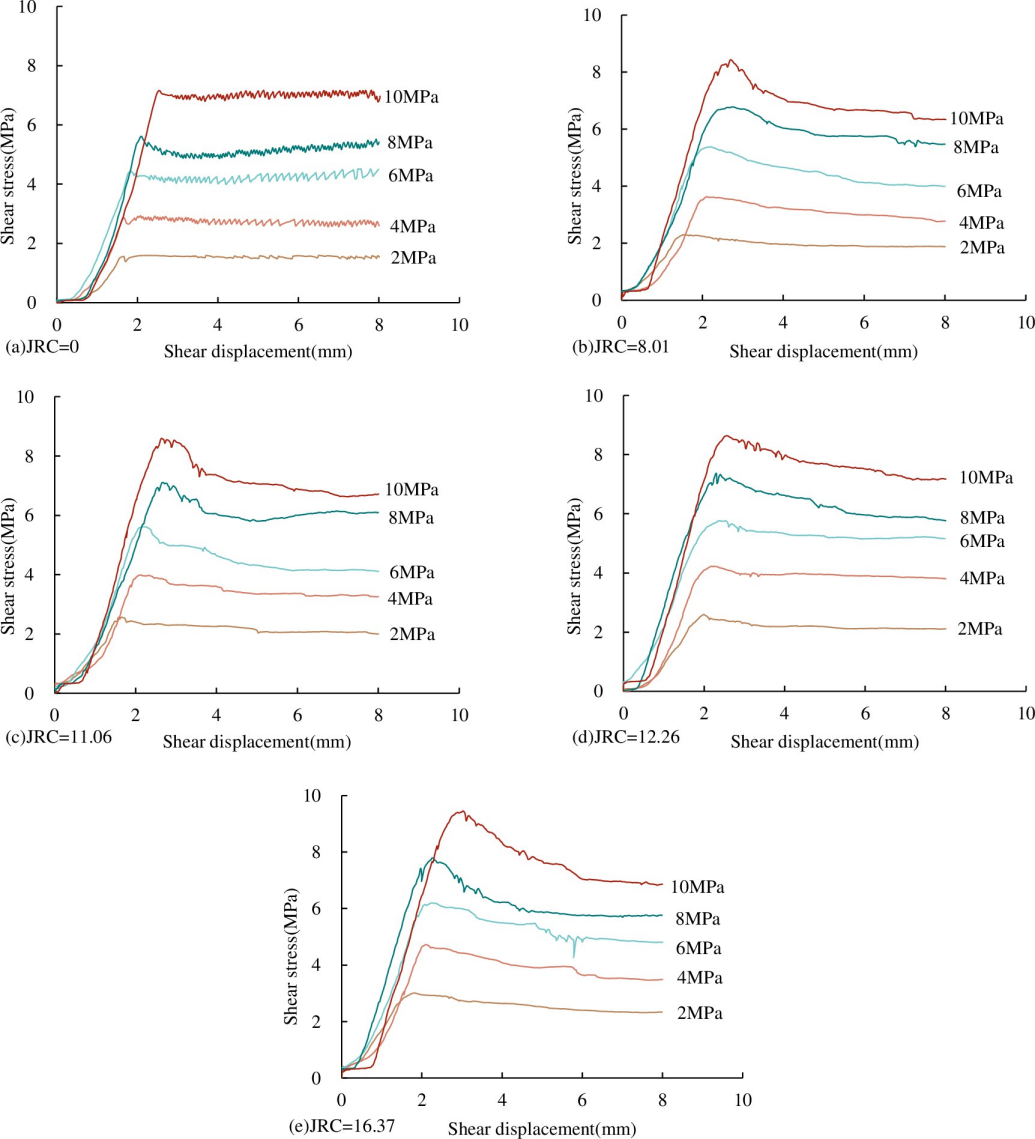
Shear stress-shear displacement curves of concrete joints with different *JRC* (a) *JRC* = 0 (b) *JRC* = 8.01 (c) *JRC* = 11.06 (d) *JRC* = 12.26 (e) *JRC* = 16.37.

The shear stress-shear displacement curves of concrete joints with different *JRC* under the CNL path are shown in Fig 8. It can be seen from Fig 8 that the shear stress-shear displacement curves of concrete joints with different *JRC* generally have a compaction stage, approximate linear stage, pre-peak yield stage, post-peak softening stage and residual stage. However, for the smooth (*JRC* = 0) joint surface, there is no post-peak softening stage in the shear stress-shear displacement curve. (1) Compaction stage. When the shear stress is applied at the beginning, the gap between the joint is pressed and sealed, the volume of the specimen is reduced, and the obvious shear shrinkage phenomenon occurs. (2) Approximate straight-line stage. After the compaction stage of the joint, the shear strength increases linearly with the increase of shear displacement. The slope of the linear growth stage represents the pre-peak shear stiffness. It can be seen from Fig 7 that the pre-peak shear stiffness increases with the increasing normal stress under the same *JRC*. (3) Pre-peak yield stage. After the end of the linear stage, the plastic yield of the specimen occurs. With the further increase of the shear displacement, the shear strength continues to increase nonlinearly, and the growth rate is getting smaller and smaller and finally reaches the peak. It can be seen from Fig 8 that under the same *JRC*, the peak shear strength increases with the increase of normal stress. (4) Post-peak softening stage. After the joint shear reaches the peak strength, the stress decreases. In the case of the same fluctuation angle, the greater the normal stress, the greater the reduction of the post-peak shear stress, and the more obvious the post-peak softening effect; in the case of the same normal stress, the larger the fluctuation angle, the greater the reduction of shear stress, and the more obvious the post-peak softening effect. (5) Residual stage. When the shear displacement continues to increase, the shear stress remains unchanged after a certain decrease, and the shear strength is called residual strength. It can be seen from the diagram that in the case of the same fluctuation angle, the residual shear strength gradually increases with the increase of normal stress.

#### 3.1.2 Analysis of shear mechanical properties

**Fig 9:**
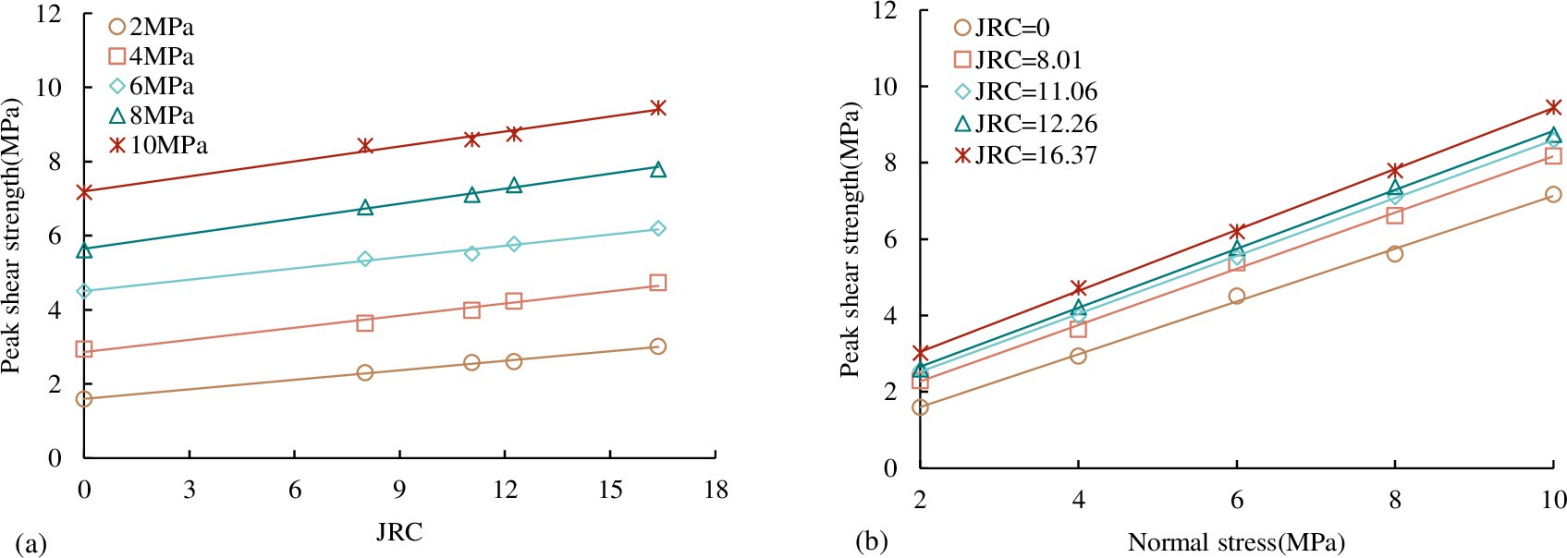
The relationship between peak shear strength and *JRC*, normal stress (a) the variation of peak shear strength with *JRC* (b) the variation of peak shear strength with normal stress.

*(1) Peak shear strength*. The relationship between the peak shear strength of concrete joints and *JRC*, normal stress under the CNL path is shown in Fig 9.

It can be seen from Fig 9 that under the same normal stress, the peak shear strength increases approximately linearly with the increase of *JRC*. Under the same *JRC*, the peak shear strength increases approximately linearly with the increase of normal stress. Under the same normal stress condition, when the *JRC* is relatively small, the height of the bulge on the joint surface is relatively large, and the contact area ratio of the upper and lower joint surfaces increases accordingly during the shear process. The bearing capacity of the joint increases and the peak shear strength increases. Similarly, under the same *JRC*, under the lower normal stress, the small protrusions of the upper and lower joint surfaces cannot be completely contacted. With the increase of the normal stress, the number of protrusion contacts increases. The larger the proportion of the contact area of the upper and lower joints, the greater the bearing capacity of the joint, and the greater the peak shear strength.

*(2) Shear strength parameters c*_*j*_ *and φ*_*j*._ It can be seen from Fig 9(B) that under the same *JRC*, the peak shear strength of the persistent joint is linearly related to the normal stress. Based on the Mohr-Coulomb criterion:

$$\tau_{p}=\sigma_{n}\;\tan\;\varphi_{j}+c_{j}$$ (3)

where *τ*_p_ is the peak shear stress, σ_n_ is the normal stress, *φ* is the internal friction angle of the joint, *c* is joint cohesion.

**Fig 10:**
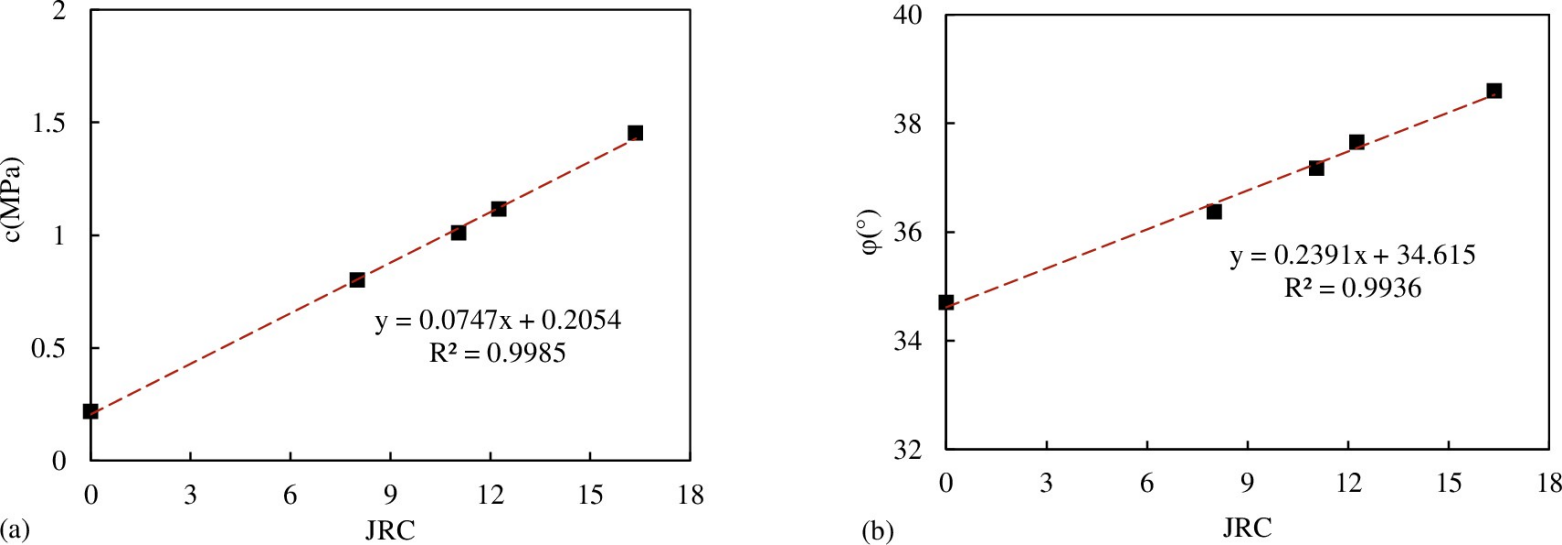
The relationship between shear strength parameters and *JRC* (a) the relationship between *c* and *JRC* (b) the relationship between internal friction angle *φ* and *JRC*.

The shear strength parameters of concrete joints with different *JRC* are obtained through the linear fitting of the results of five joint specimens with different *JRC*, as shown in Table 4. According to the results of Table 4, we can get the relationship between the comprehensive shear strength parameter joint internal friction angle *φ* and joint cohesion *c* and *JRC* as shown in Fig 10. From Fig 10, it can be seen that the comprehensive shear strength parameter internal friction angle *φ* and joint cohesion c increase linearly with the increase of *JRC*.

**Table 4:** Fig 9(b) in the straight-line fitting results.

*JRC*	*τ*_p_ = *σ*_n_ tan *φ*_j_+c_j_		*R* ^2^
	*φ*_j_	*c*_j_	
0	34.7	0.2166	0.92
8.01	36.37	0.8005	0.99
11.06	37.17	1.0084	0.97
12.26	37.65	1.1152	0.95
16.37	38.59	1.4517	0.99

**Fig 11:**
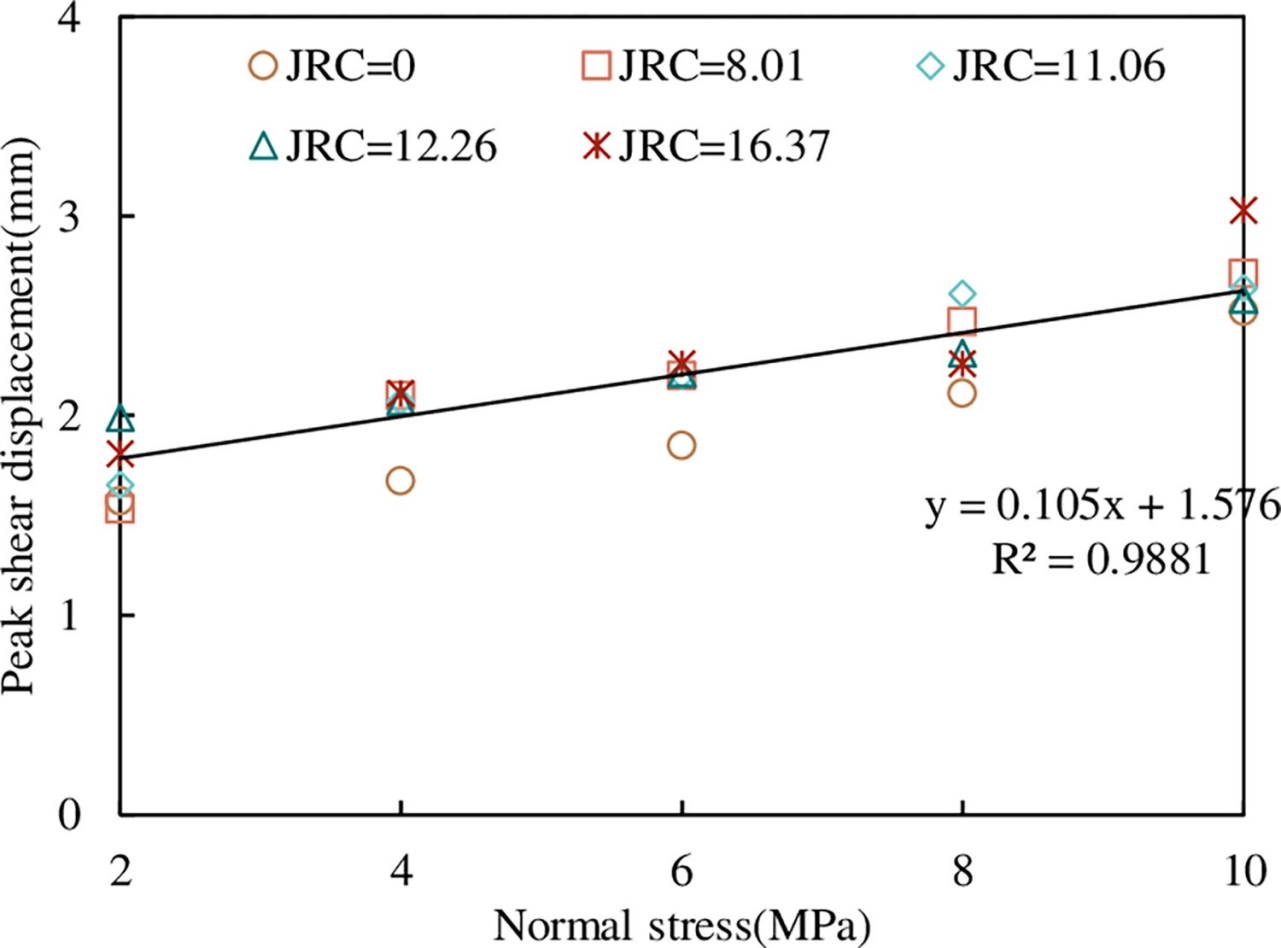
Relationship between peak shear displacement and normal stress.

*(3) Peak shear displacement*. From the shear stress-shear displacement curve in Fig 8, we can see that under the same *JRC*, with the increase of normal stress, the shear displacement corresponding to the peak shear strength of the joint becomes larger and larger. The relationship between peak shear displacement and normal stress of concrete joints under the CNL path is shown in Fig 11.

It can be seen from Fig 11 that the peak shear displacement increases linearly with the increase of normal stress, and is not greatly affected by *JRC*, indicating that the peak shear displacement is more sensitive to normal stress, which is consistent with the conclusion of Wibowo [47]. The Wibowo formula is as follows:

$$u_{p}=a+b\sigma_{n}$$ (4)

where *u*_p_ is the peak shear displacement, σ_n_ is the normal stress, and a and b are the fitting parameters. By averaging the peak shear displacement of different *JRC* concrete joints under the same normal stress, the relationship between the peak shear displacement and the normal stress can be obtained. By fitting, a = 1.576, b = 0.105 in formula (4) can be obtained. From this, we can obtain the prediction formula of the peak shear displacement of concrete joints:

$$u_{p}=1.576+0.105\sigma_{n}$$ (5)

To quantify the error of Formula (5), the average relative error is used to evaluate its applicability [23], the expression of the average relative error is given as follows:

$$\delta =\frac{1}{n}\sum_{j=1}^{n}\left|\frac{u_{pj}-u_{ij}}{u_{pj}}\right|\times 100\%$$ (6)

where, *δ* is the average relative error, %; *u*_*pj*_ is the peak shear displacement test value, MPa; *u*_*ij*_ is the predicted value of peak shear displacement; *n* is the number of tests. The number of tests in this paper is 25 times, and the average relative error is 6.7%.

*(4) Pre-peak shear stiffness*. The slope of the approximate straight-line segment before the peak of the shear stress-shear displacement curve can be expressed as the shear stiffness before the peak [41], which is a parameter reflecting the ability of the joint to resist shear deformation. For concrete joint specimens with different *JRC*, under the same normal stress condition, when the *JRC* is small, the protrusion of the joint is small, the climbing is relatively easy, the ability of the joint to resist shear deformation is small, and the shear stiffness before the peak of the joint is also small. With the increase of *JRC*, the protrusion of the joint increases, the difficulty of pre-peak climbing increases, the ability of a joint to resist shear deformation increases, and the pre-peak shear stiffness of the joint also increases. Under the same *JRC*, when the normal stress is low, the upper and lower joint surfaces cannot be completely contacted, so the ability to resist shear deformation is relatively small. With the increase of normal stress, the contact area ratio of the upper and lower joint surfaces increases, the match effect of the upper and lower joint surfaces increases, and the bearing capacity of the joint also increases, so the ability to resist shear deformation also increases accordingly.

**Fig 12:**
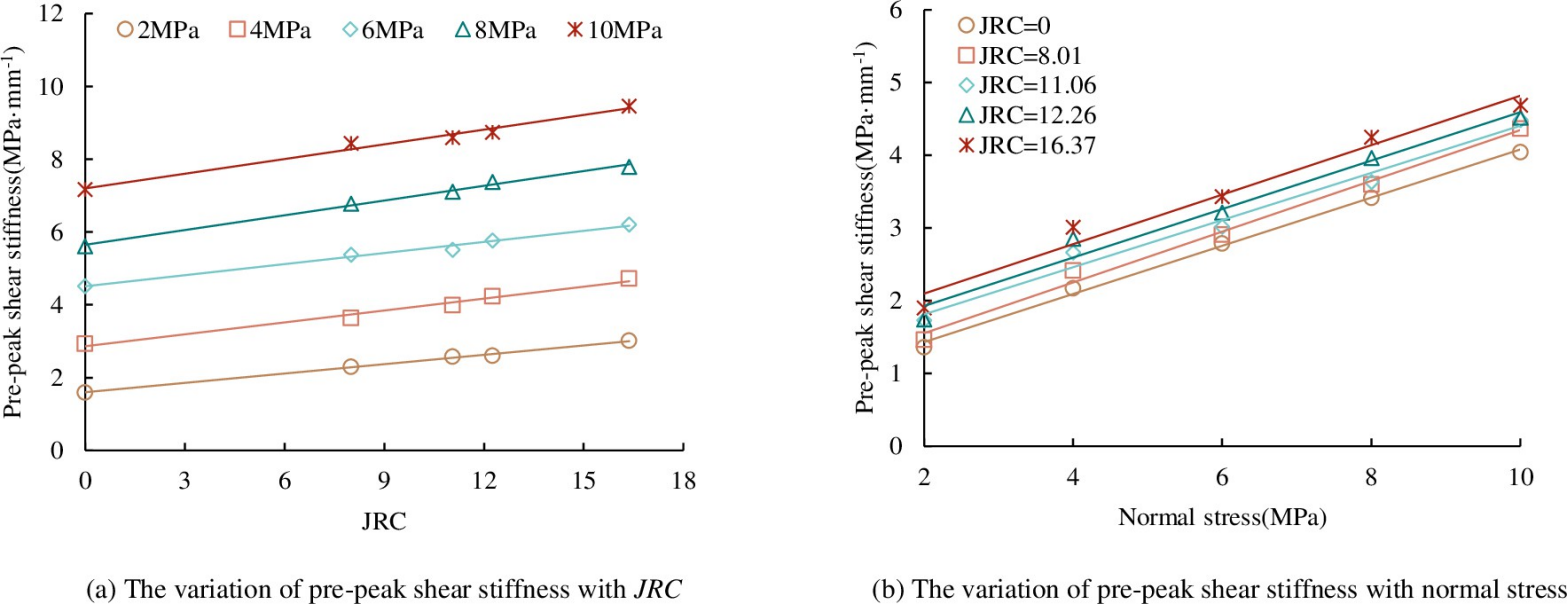
The relationship between pre-peak shear stiffness and *JRC*, normal stress. (a) The variation of pre-peak shear stiffness with *JRC*. (b) The variation of pre-peak shear stiffness with normal stress.

The relationship between pre-peak shear stiffness and *JRC* is shown in Fig 12(A). It can be seen from Fig 12(A) that the pre-peak shear stiffness increases with the increase of *JRC*. The linear relationship between pre-peak shear stiffness and *JRC* can be obtained by fitting the pre-peak shear stiffness of concrete joints under the same normal stress. With the increase of *JRC*, the pre-peak shear stiffness increases linearly. The relationship between the pre-peak shear stiffness and the normal stress of the concrete joint is shown in Fig 12(B). It can be seen from Fig 12(B) that the pre-peak shear stiffness increases with the increase of the normal stress. By fitting the pre-peak shear stiffness of concrete joints with the same undulating angle, the linear relationship between pre-peak shear stiffness and normal stress can be obtained. With the increase of normal stress, the pre-peak shear stiffness increases linearly.

**Fig 13:**
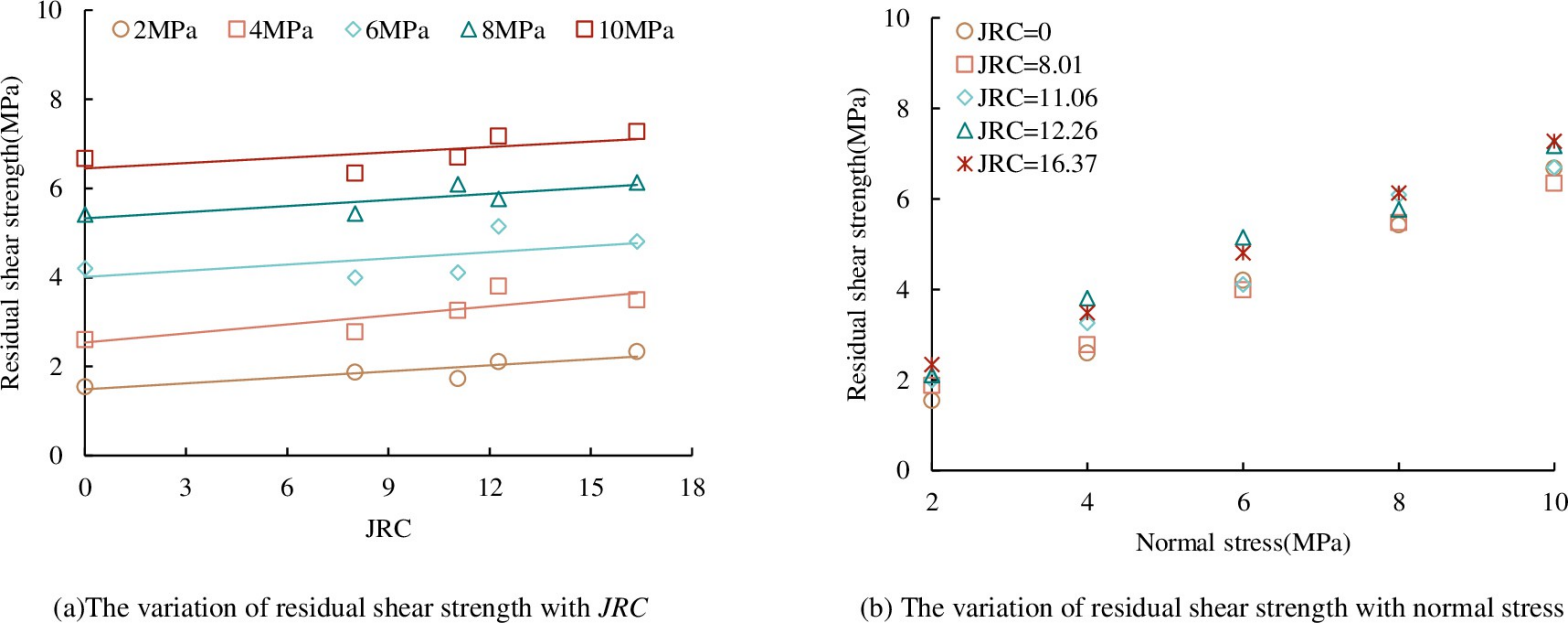
The relationship between residual shear strength and *JRC*, normal stress. (a) The variation of residual shear strength with *JRC*. (b) The variation of residual shear strength with normal stress.

*(5) Residual shear strength*. From the shear stress-shear displacement curve in Fig 8, it can be seen that under the same *JRC*, the residual shear strength increases with the increase of normal stress. The relationship between the residual shear strength of concrete joints and *JRC* and normal stress under the CNL path is shown in Fig 13. It can be seen from Fig 13 that the residual shear strength increases linearly with the increase of *JRC* and normal stress. This is consistent with the law of peak shear strength and *JRC*, normal stress in Fig 9. In the residual wear stage, although most of the bulges of the joints have been cut off, there are still tiny bulges on the joint that have not been destroyed. These tiny bulges have a certain bearing effect in the residual stage. Under the same *JRC*, with the increase of normal stress, the proportion of the remaining small convex contact area of the upper and lower joint surfaces in the residual stage increases, and the bearing capacity of the joint also increases, so the residual shear strength also increases accordingly. Under the same normal stress condition, with the increase of *JRC*, the remaining small protrusions on the upper and lower joint surfaces in the residual stage increase accordingly, the bearing capacity of the joint increases, and the residual shear strength increases.

#### 3.1.3 Analysis of joint failure morphology characteristics

**Fig 14:**
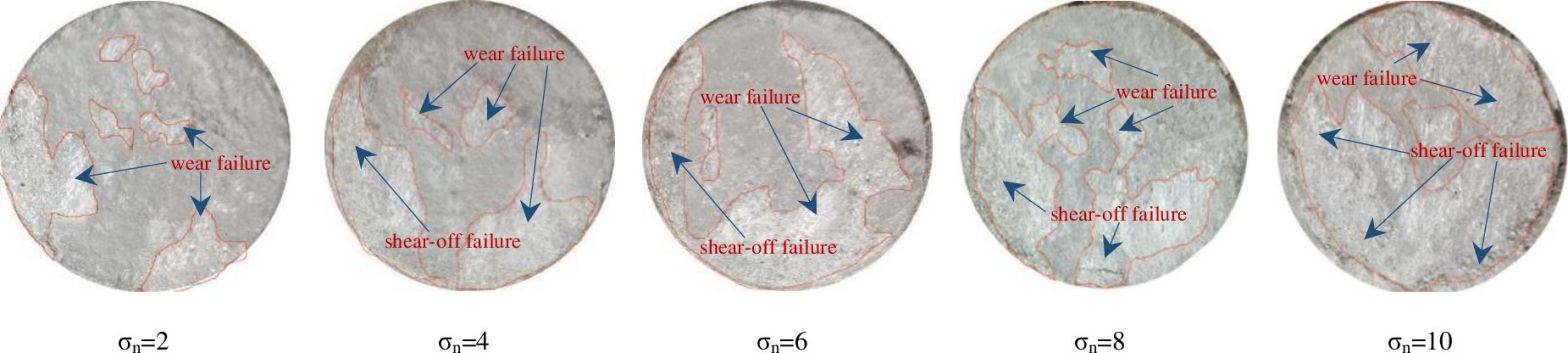
Failure modes of the joint surface under different normal stresses of group C specimens under the CNL path.

When the rock joint is damaged by shear force, most of the joint convex parts are wear and shear-off failure. The joint specimens will show different damage degrees and forms under different normal stresses. Under the same *JRC* (*JRC* = 11.06), the failure morphology of concrete joint surface under different normal stresses is shown in Fig 14. From Fig 14, we can see that when the normal stress is small, the failure of the joint surface is mainly dominated by wear failure, and the area of wear failure is relatively small. This is because, under the condition of low normal stress, the contact area of the upper and lower joint surfaces is relatively small, so the area of the shear failure area is relatively small; at the same time, the match effect of the upper and lower joint surfaces is small, and it is more inclined to climbing wear damage when sheared. With the increase of normal stress, we can see that the area of the joint surface failure area increases significantly. At the same time, the joint surface has an obvious shear failure, which is no longer a single wear, and the small block debris can be seen in the sheared debris. This is because with the increase of normal stress, on the one hand, the contact area of upper and lower joint surfaces increases, so the proportion of failure area increases; on the other hand, with the increase of normal stress, the match effect of the upper and lower joint surfaces is enhanced. When sheared, the convex is cut directly from the root, and no climbing occurs. It can be seen from the morphology of the joint surface damage that the edge damage of the joint is relatively serious, which is mainly caused by stress concentration. When the normal stress is small, the stress concentration phenomenon is relatively small. With the increase of the normal stress, the stress concentration phenomenon is relatively obvious, and the damage to the joint edge is more serious.

### 3.2 Shear test results and analysis under the UNLCSL path

Studying the characteristics of the stress-time curve is conducive to a more intuitive understanding of the stress of the direct shear test under the UNLCSL path, and can also accurately determine the unloading instability time from the stress-time curve. Therefore, the stress-time curve characteristics of the two stages of shear loading and normal unloading are studied.

Before the analysis of shear mechanics parameters, we first define some related parameters. the instability shear strength τ_*i*_: the shear stress corresponding to the shear instability moment of the joint in the normal unloading stage; instability normal stress σ_*i*_: in the normal unloading stage, the normal stress corresponding to the moment when the shear instability occurs on the joint; theoretical shear strength τ_t_: based on the strength law obtained by the CNL path, the shear strength obtained by inverse calculation according to σ_*i*_; the normal stress unloading amount Δσ_n_: the absolute value of the difference between σ_*i*_ and σ_1_.

#### 3.2.1 Characteristics of stress-time curve

**Fig 15:**
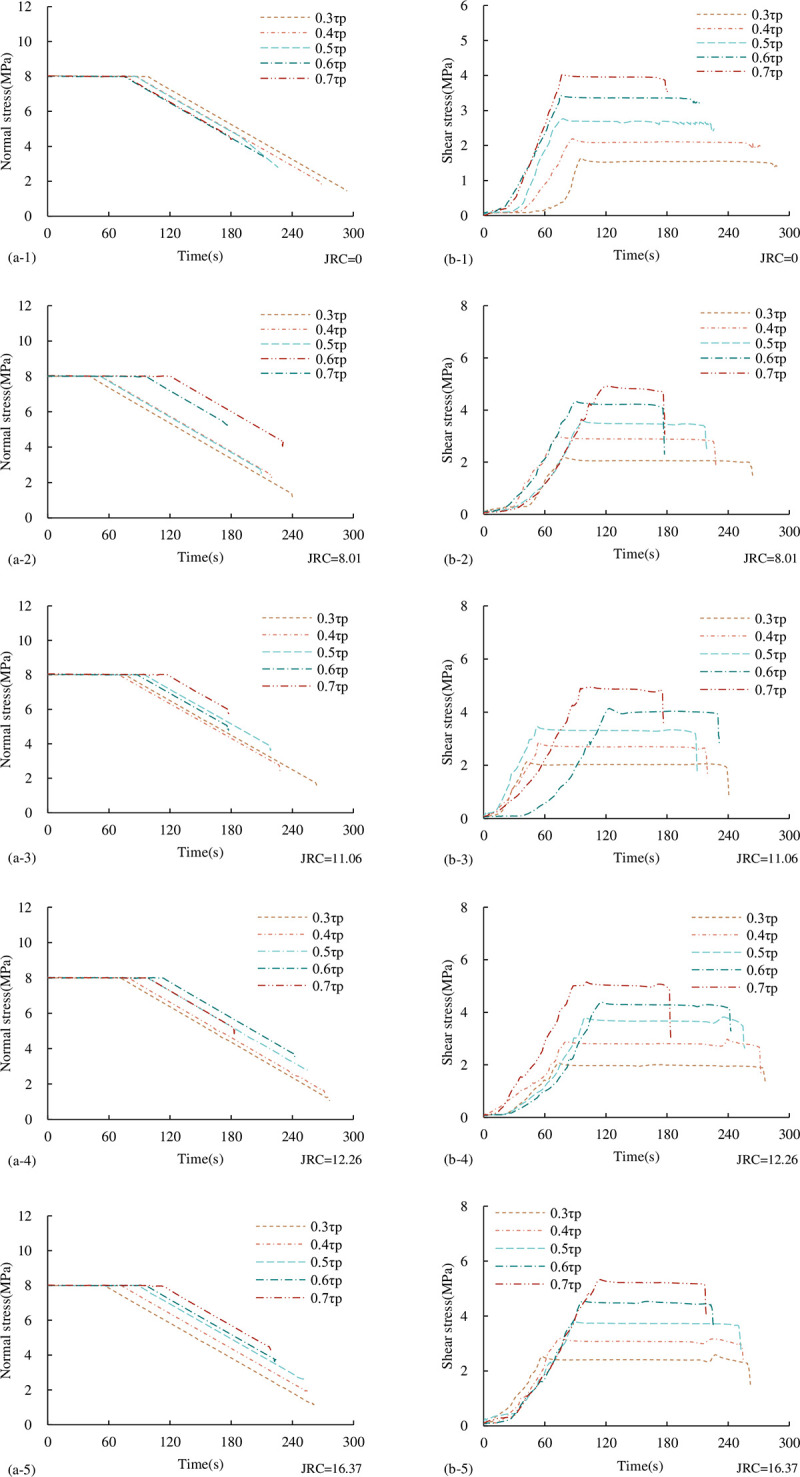
Stress-time curve under the UNLCSL path (a) Normal stress-time curve (b) Shear stress-time curve.

Fig 15 shows the shear stress-time curves of through-joints with different *JRC* under the UNLCSL path (excluding the first step of normal loading, only corresponding to the second step, the third step of shear loading and normal unloading process). As can be seen from Fig 15:

The shear loading stage: after σ_1_ loading is completed, the shear loading stage is entered, and the normal stress remains constant at this stage. The shear stress gradually increases with time. Because the shear direction adopts the displacement loading method, it is similar to the shear stress-displacement curve of the CNL path. When the shear stress is applied at the beginning, the gap between the joint is pressed and closed. Therefore, the shear stress-time curve has an obvious compaction stage, and then it increases in a nearly linear segment. When the shear stress reaches the τ_1_ setting value, it remains constant, and the normal unloading begins.

The normal unloading stage: the normal stress decreases uniformly with time until the decrease of the normal stress causes the shear-bearing capacity of the joint to be less than the set τ_1_, and the joint undergoes instability shear failure. Currently, the normal stress no longer decreases uniformly with time but drops directly. At the beginning of the normal unloading, the shear stress will drop slightly, and then remain basically constant. When the shear bearing capacity of the joint is less than the set τ_1_ due to the decrease of the normal stress, the joint will undergo instability shear failure. At this time, the shear stress will no longer remain unchanged, but drop instantaneously. Therefore, we can accurately judge the shear instability point of concrete joints during normal unloading by the stress-time curve.

In addition, it can be seen from Fig 15 that with the increase of τ_1_, the time of shear loading increases and the time of normal unloading decreases. This is because the greater the τ_1_, the greater the ability to resist shear deformation required for instability and failure. On the one hand, the ability to resist deformation is determined by the material itself. On the other hand, the greater the normal load, the larger the contact area ratio of the joint, the greater the ability of the joint to resist deformation. Therefore, for the joint of the same material and *JRC*, the shear resistance of the joint is proportional to the normal stress. The greater τ_1_, the greater τ_*i*_, the greater σ_*i*_, and the shorter the time required for unloading instability at the same σ_1_ and unloading rate.

#### 3.2.2 Characteristics of displacement-time curve

**Fig 16:**
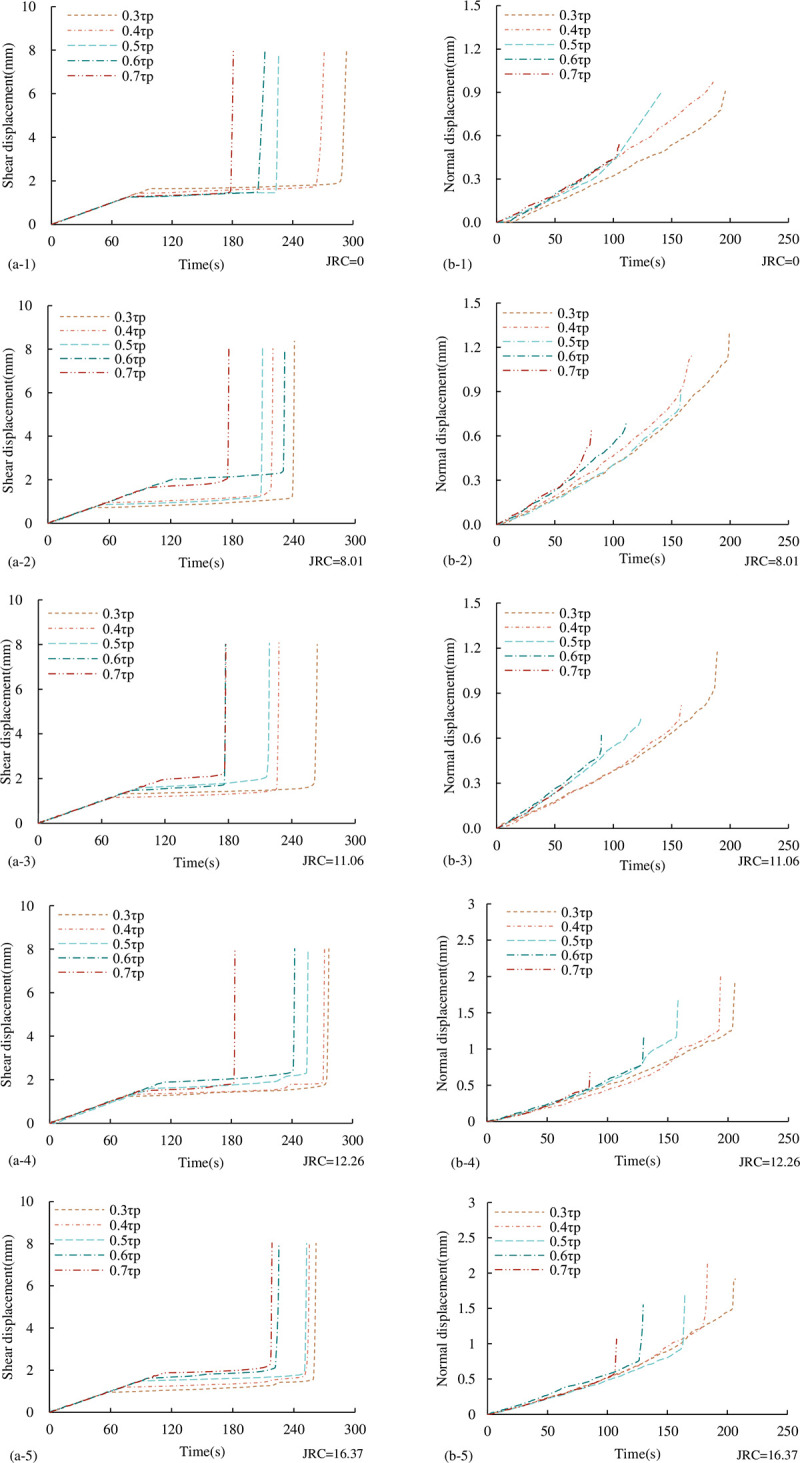
Displacement-time curve under the UNLCSL path (a) shear displacement-time curve (b) normal displacement-time curve.

In addition to the sudden change of stress during shear instability failure, the displacement will also change abruptly at the time of instability. Therefore, the time of instability can also be accurately judged by the displacement-time curve. Under the UNLCSL path, the displacement-time curves of persistent joints with different *JRC* under different τ_1_ are shown in Fig 16. The shear displacement-time curve only corresponds to the second step of the shear loading process and the third step of normal unloading process, and the normal displacement-time curve only corresponds to the third step of the normal unloading process. It can be seen from Fig 16: shear loading stage: after σ_1_ loading is completed, the shear loading is started, and the shear displacement increases uniformly with the increase of time. The normal unloading stage: after the normal unloading begins, the shear load remains constant, and the displacement remains constant. Until a certain moment, when the shear capacity of the joint surface is not enough to resist τ_1_, the shear instability failure occurs, and the shear displacement increases instantaneously. In the normal unloading stage, before the instability failure, the normal displacement gradually increases with time. On the one hand, it is caused by the normal rebound caused by the normal unloading, and on the other hand, it is caused by the dilatancy effect caused by the shear failure. At the same time, we can also see that the larger τ_1_, the smaller the normal displacement during instability. This is because the larger τ_1_, the larger the normal stress during instability, the smaller Δσ_n_, and the shorter the unloading time, so the normal rebound is relatively small.

#### 3.2.3 Shear stress-shear displacement curve characteristics

**Fig 17:**
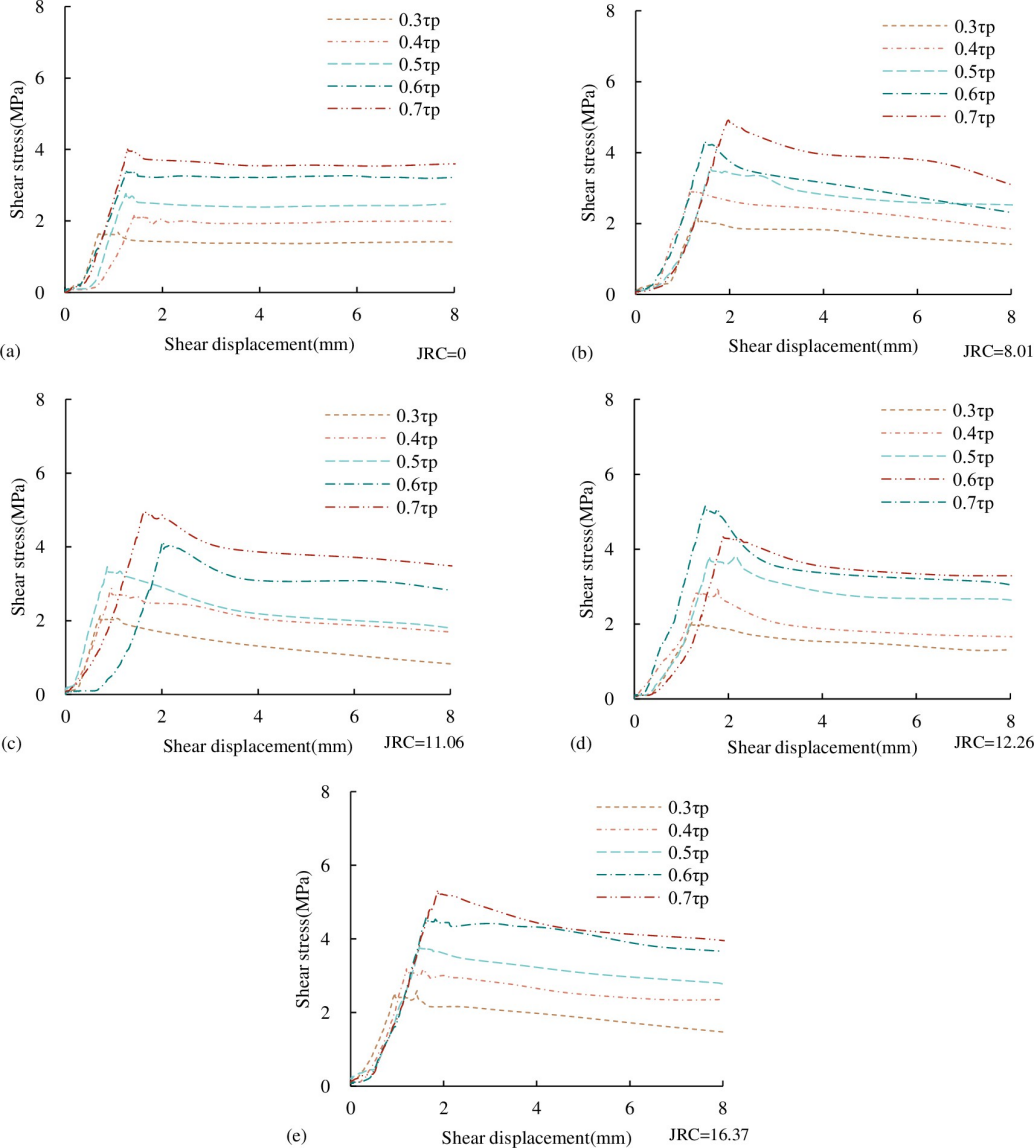
Shear stress-shear displacement curve under the UNLCSL path (a) *JRC* = 0 (b) *JRC* = 8.01 (c) *JRC* = 11.06 (d) *JRC* = 12.26 (e) *JRC* = 16.37.

The shear stress-shear displacement curves of different *JRC* persistent joints under UNLCSL conditions under different τ_1_ conditions are shown in Fig 17. The peak point of the shear stress-displacement curve in Fig 17 represents the start time of normal unloading, not the time of joint failure. Before the peak point, it belongs to the shear loading stage. Similar to the shear stress-shear displacement curve of the CNL path in Fig 8, the shear stress-displacement curve under the UNLCSL path also has an obvious compaction section and an approximate straight section. Since the ability of the joint to resist deformation is not limited at the beginning of the normal unloading stage, there is no obvious pre-peak yield section. At the beginning of the normal unloading, the shear stress decreased slightly, and then remained constant. As the normal unloading stage begins, the normal stress gradually decreases, the match effect of the upper and lower joint surfaces is weakened, and the bearing capacity of the joint is reduced. At a certain moment, the bearing capacity of the joint is not enough to resist the constant shear stress, and the joint is instability. The instability point can be judged by the stress-time and displacement-time curves.

#### 3.2.4 Analysis of shear mechanical parameters

Through the analysis and calculation of the test results, we get the relevant experimental data as shown in Table 5.

**Table 5:** Test results under the UNLCSL path.

*JRC*	Specimen number	τ_*i*_ /MPa	σ_*i*_ /MPa	τ_t_ /MPa	σ_1_ /MPa	Δσ_n_ /MPa
0	ⅡA-0.3	1.43	1.81	1.47	8	6.19
	ⅡA-0.4	1.84	2.35	1.84	8	5.65
	ⅡA-0.5	2.41	3.16	2.40	8	4.84
	ⅡA-0.6	2.91	3.89	2.91	8	4.11
	ⅡA-0.7	3.68	4.89	3.60	8	3.11
8.01	ⅡB-0.3	1.70	1.47	1.88	8	6.53
	ⅡB-0.4	2.41	2.46	2.61	8	5.54
	ⅡB-0.5	2.87	2.97	2.99	8	5.03
	ⅡB-0.6	3.84	4.36	4.01	8	3.64
	ⅡB-0.7	4.78	5.47	4.83	8	2.53
11.06	ⅡC-0.3	2.07	1.80	2.12	8	6.20
	ⅡC-0.4	2.93	2.78	2.87	8	5.22
	ⅡC-0.5	3.47	3.94	3.77	8	4.06
	ⅡC-0.6	4.21	4.55	4.24	8	3.45
	ⅡC-0.7	4.71	5.23	4.76	8	2.77
12.26	ⅡD-0.3	1.82	1.41	2.20	8	6.59
	ⅡD-0.4	2.64	2.46	3.01	8	5.54
	ⅡD-0.5	3.02	2.78	3.26	8	5.22
	ⅡD-0.6	3.93	4.31	4.44	8	3.69
	ⅡD-0.7	4.97	5.25	5.17	8	2.75
16.37	ⅡE-0.3	1.92	1.25	2.45	8	6.75
	ⅡE-0.4	2.63	1.62	2.74	8	6.38
	ⅡE-0.5	3.46	2.79	3.68	8	5.21
	ⅡE-0.6	4.18	3.74	4.44	8	4.26
	ⅡE-0.7	4.88	4.47	5.02	8	3.53

**Fig 18:**
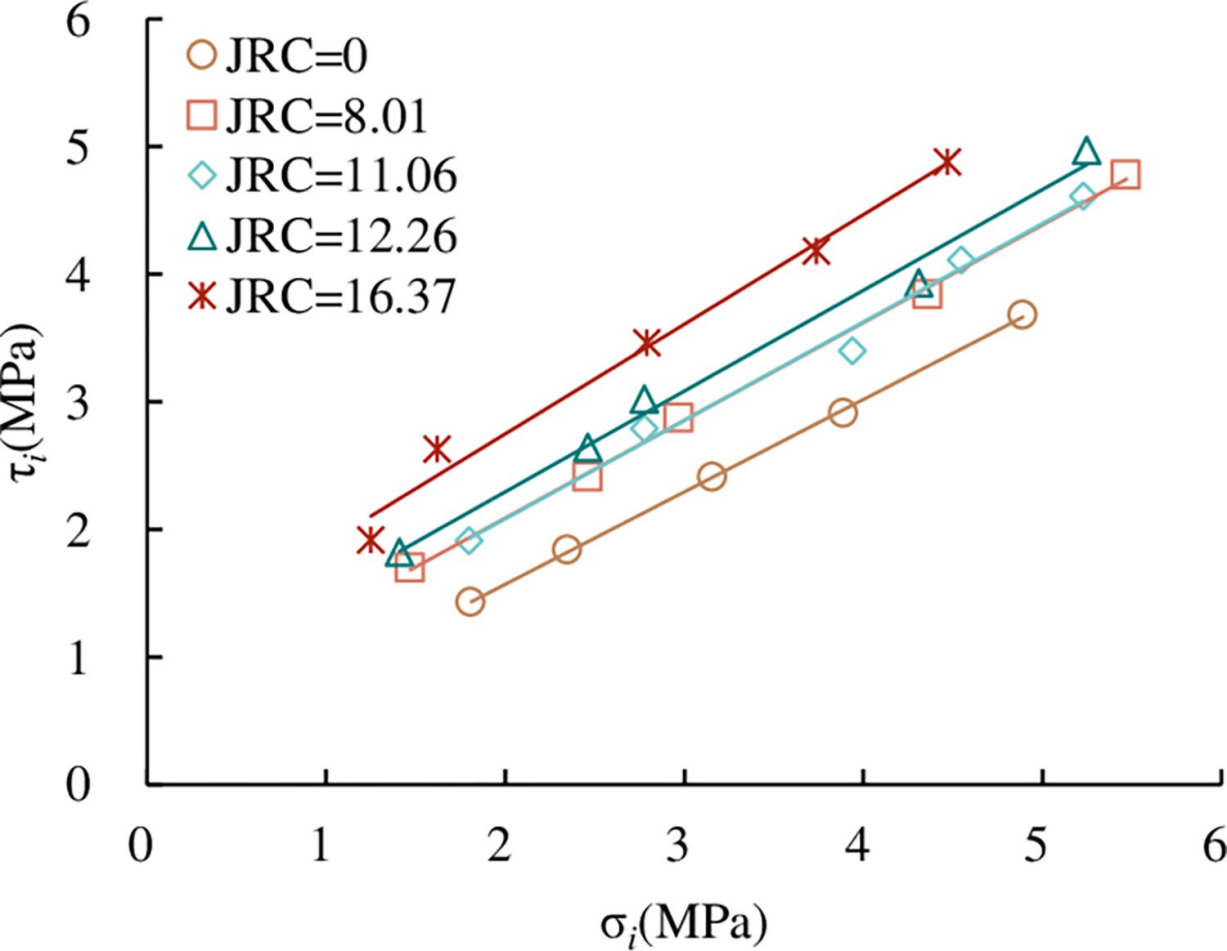
Relationship between τ_*i*_ and σ_*i*_.

*(1) Relationship between τ*_*i*_ *and σ*_*i*_. According to the test results in Table 5, the relationship between τ_*i*_ and σ_*i*_ is shown in Fig 18. From Fig 18, shows that τ_*i*_ increases linearly with σ_*i*_. At the same time, we can see that under the same σ_*i*_, τ_*i*_ increases with the increase of *JRC*. This is the same as the law of peak shear strength and normal stress in direct shear tests under the CNL path, which is not explained here.

*(2) Shear strength parameters of persistent joints under the UNLCSL path*. Similar to the CNL path, it can be seen from Fig 18 that τ_*i*_ of the persistent joint is linearly related to σ_*i*_ under the same *JRC*, which conforms to the Mohr-Coulomb criterion. Through the linear fitting of the test results of five joint specimens with different *JRC*, the shear strength parameters of joints with different *JRC* under normal unloading are obtained as shown in Table 6.

**Table 6:** Fig 18 in each curve fitting results.

*JRC*	*τ*_*s*_ = *σ*_*s*_ tan *φ*_*s*_+*c*_*s*_		*R* ^2^
	*φ*_*s*_	*c*_*s*_	
0	35.9	0.123	0.99
8.01	37.43	0.5589	0.99
11.06	37.65	0.5404	0.98
12.26	38.28	0.7171	0.98
16.37	40.58	1.0323	0.98

**Fig 19:**
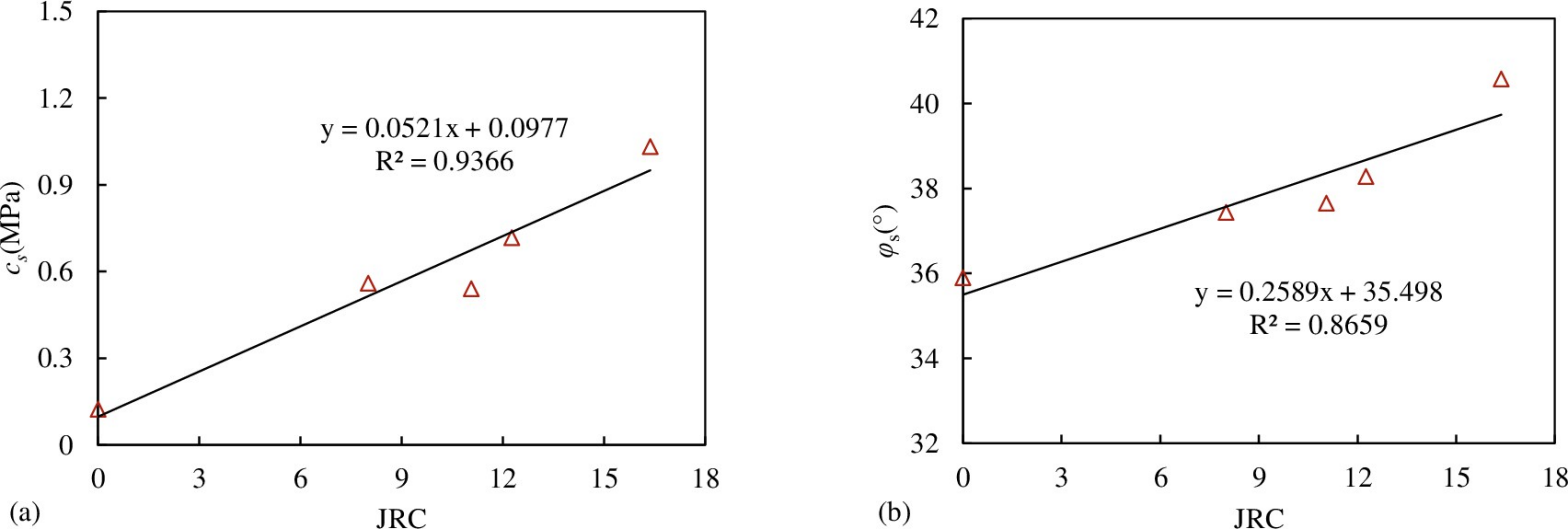
The relationship between the shear strength parameters and *JRC* under the UNLCSL path (a) the relationship between *c*_s_ and *JRC* (b) the relationship between φ_s_ and *JRC*.

According to the results of Table 6, we can get the shear strength parameters of joints with different *JRC* under the UNLCSL path. The relationship between joint internal friction angle *φ*_s_, joint cohesion *c*_s_ and *JRC* is shown in Fig 19. It can be seen from Fig 19 that the shear strength parameters of joints with different *JRC* under the UNLCSL path increase linearly with the increase of *JRC*.

**Fig 20:**
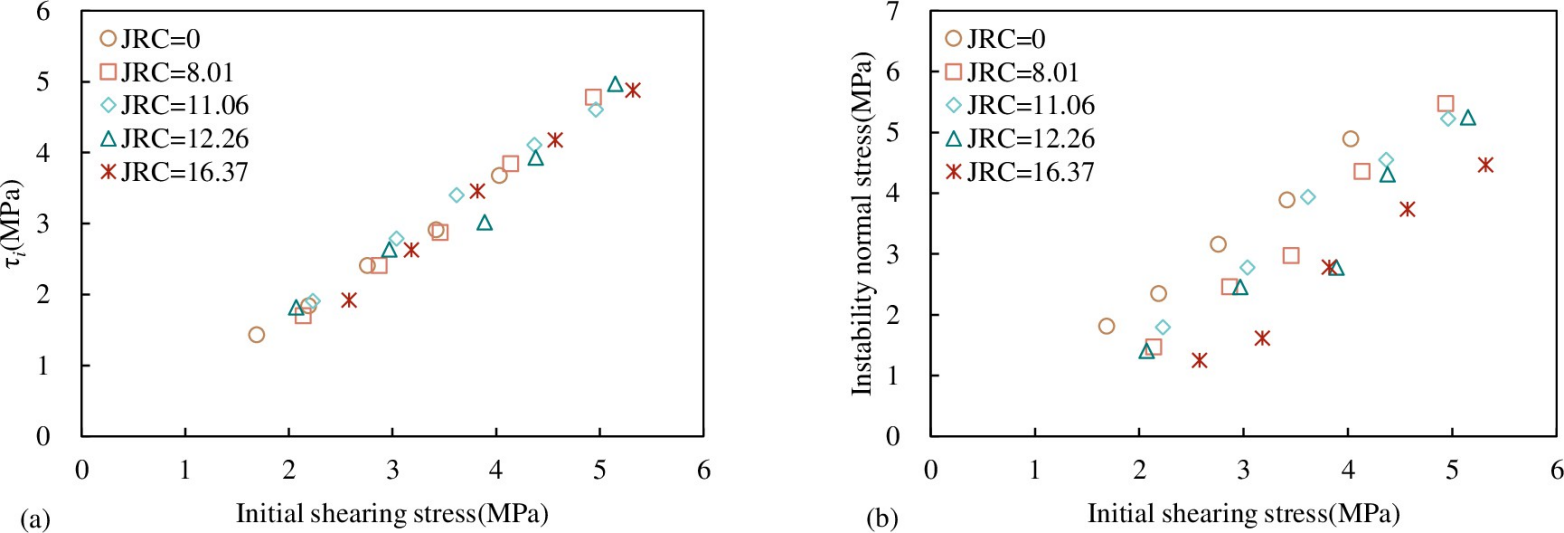
The relationship between the τ_1_ and τ_*i*_, σ_*i*_ (a) the relationship between τ_*i*_ and τ_1_ (b) the relationship between σ_*i*_ and τ_1_.

*(3) The relationship between τ*_*i*_, *σ*_*i*_ *and τ*_*1*_. Fig 20 lists the relationship between τ_*i*_, σ_*i*_ and τ_1_. It can be seen from Fig 20(A) that the greater τ_1_, the greater σ_*i*_, and the greater τ_*i*_. It can be seen from Fig 20(B) that under the same *JRC*, the instability of normal stress increases with the increase of τ_1_. The larger τ_1_ is, the greater τ_*i*_ is. According to the Mohr-Coulomb criterion, the normal stress corresponding to instability is greater. Under the same τ_1_, the larger the *JRC*, the smaller σ_*i*_; under the same τ_1_, τ_*i*_is equivalent, and τ_*i*_is positively correlated with the normal stress and *JRC*. The larger the *JRC*, the smaller the instability of normal stress.

**Fig 21:**
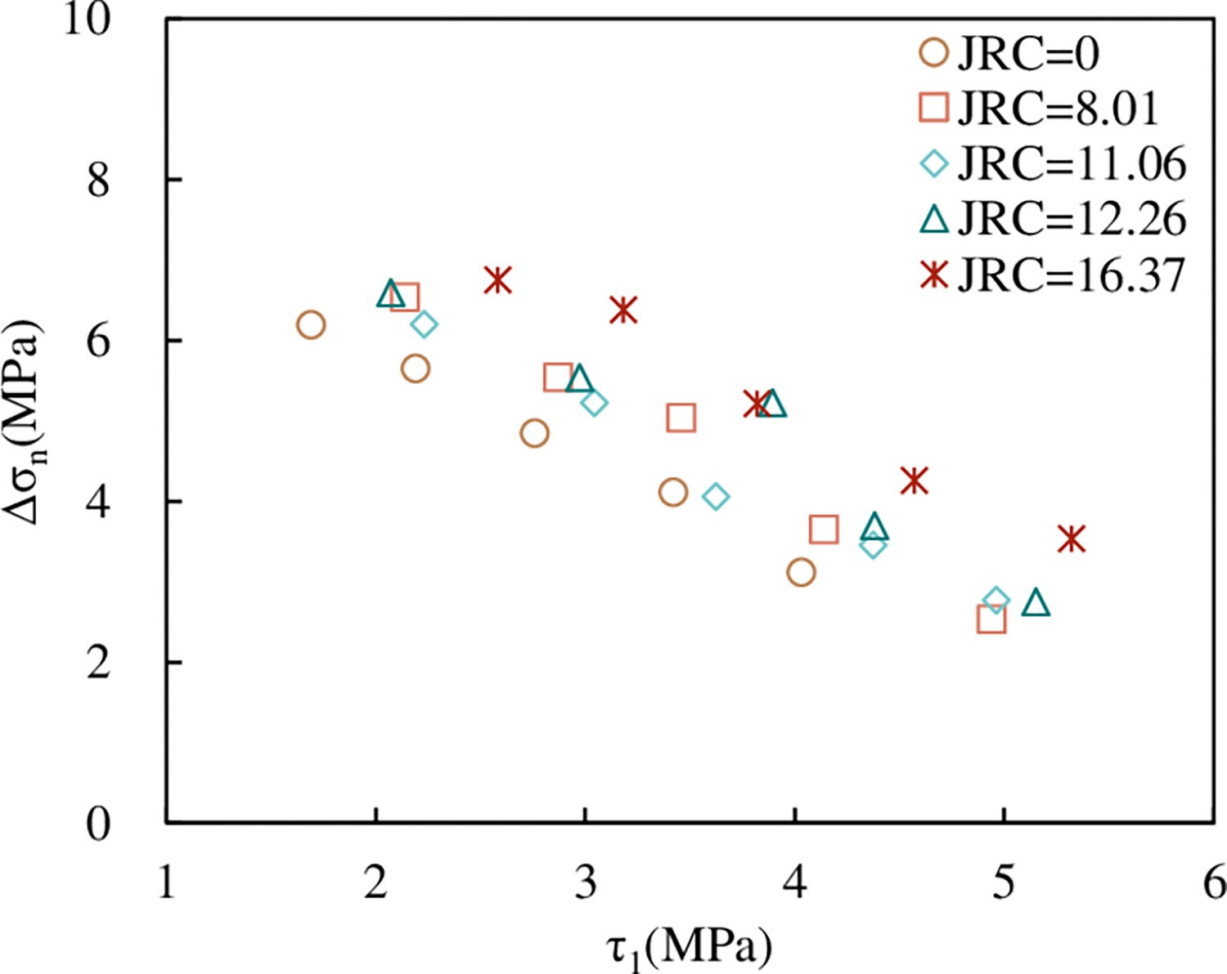
The relationship between Δσ_n_ and τ_1_, *JRC*.

*(4) The relationship between Δσ*_*n*_ *and τ*_*1*_, *JRC*. Fig 21 shows the relationship between Δσ_n_ and τ_1_ under normal unloading conditions. It can be seen from Fig 21 that Δσ_n_ decreases linearly with the increase of τ_1_. The greater the τ_1_, the greater τ_*i*_. According to the Mohr-Coulomb criterion, the normal stress corresponding to instability increases with the increase of τ_*i*_. For the same σ_1_, the greater the normal stress during instability, the shorter the normal unloading time, that is, the smaller Δσ_n_. In addition, we can also see that under the same τ_1_, the larger the *JRC*, the greater the corresponding Δσ_n_; this corresponds to Fig 20(B). The larger the *JRC* is, the smaller σ_*i*_ is, and the larger Δσ_n_ is.

#### 3.2.5 Analysis of joint failure morphology characteristics

**Fig 22:**
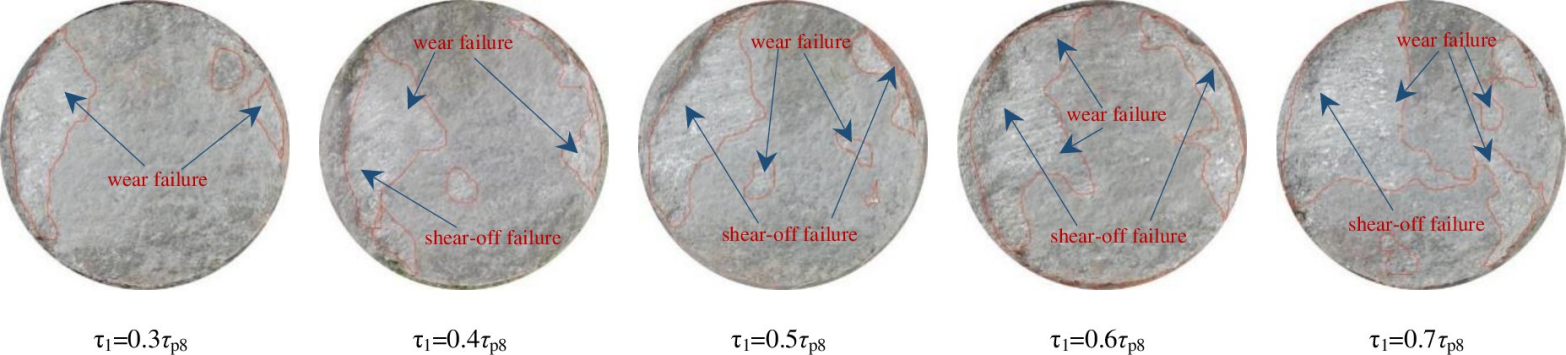
Failure modes of joint surfaces under different τ_1_of group C specimens under the UNLCSL path.

When the rock joint is damaged by shear force, most of the joint convex parts are wear and shear-off failure. The joint specimens will show different damage degrees and failure forms under different τ_1_. The failure morphology of the joint with a *JRC* of 11.06 under different τ_1_is shown in Fig 22.

It can be seen from Fig 22 that when τ_1_ is small, the area of joint surface failure is relatively small, the particle size of the debris obtained by shearing is particularly small, and the joint surface failure is mainly dominated by wear failure. This is because under the condition of small τ_1_, the resistance of a small bulge is small and directly destroyed, but the resistance of a large bulge is relatively large, and it can reach the limit of bearing capacity, so only a small part is destroyed. With the increase of τ_1_, it can be seen that the area of the joint surface failure area increases significantly. At the same time, small pieces of debris can be seen in the sheared debris. This is because with the increase of τ_1_, the joint surface has obvious shear failure, which is no longer a single wear, and the small protrusions of the upper and lower joint surface damage increase, so the proportion of the damage area increases. It can be seen from the morphology of the joint surface damage that the edge damage of the joint surface is relatively serious, which is mainly caused by stress concentration. When τ_1_ is small, the stress concentration phenomenon is relatively small. With the increase of normal stress, the stress concentration phenomenon is relatively obvious, and the damage to the joint edge is more serious.

### 3.3 Shear test results and analysis under the path of UNLISL/UNLUSL

#### 3.3.1 Characteristics of stress-time curve

**Fig 23:**
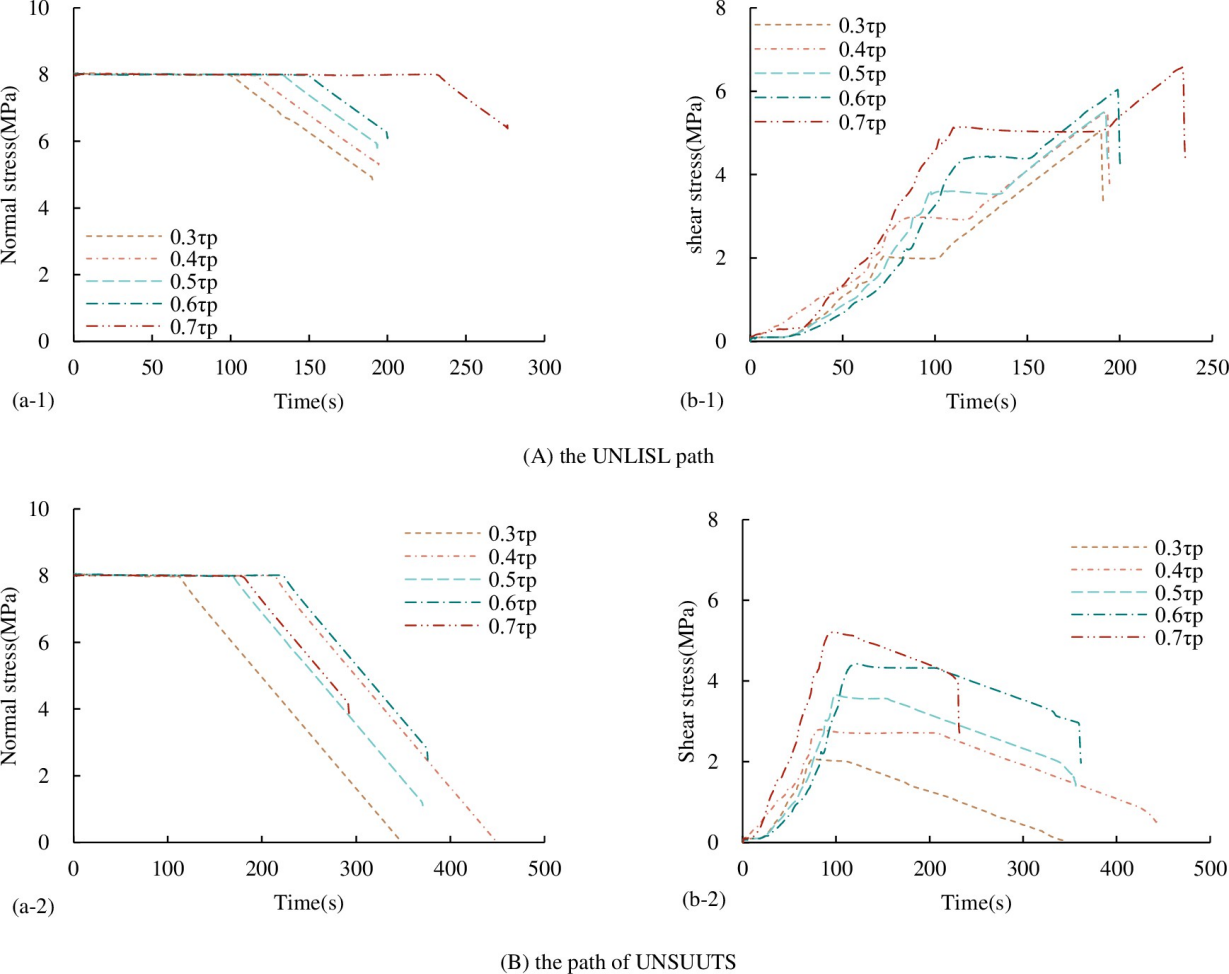
Stress-time curves under different unloading stress paths (a) normal stress-time curves (b) shear stress-time curves.

The stress-time curve of concrete joints under the UNLISL/UNLUSL path is shown in Fig 23 (excluding the first step of normal loading, only corresponding to the second step and the third step of shear loading, and unloading process). The normal stress-time curve Fig 23(A) under the UNLISL path is similar to that under the UNLCSL path, and the shear stress-time curve is different. At the beginning of normal unloading, the shear stress increases linearly with the increase of time (the loading rate is constant), until the decrease of normal stress causes the shear-bearing capacity of the joint to be less than the corresponding shear stress at this time, the joint occurs instability shear failure. At this time, the shear stress no longer increases uniformly with time but falls directly. At this time, it is the joint shear instability point under the stress path.

The normal stress-time curve Fig 23(B) under the UNLUSL path is similar to that under the UNLCSL path, and the shear stress-time curve is similar to that under the UNLISL path. The only difference is that at the beginning of the normal unloading, the shear stress decreases linearly with the increase of time (the unloading rate is constant) until the decrease of the normal stress causes the shear bearing capacity of the joint to be less than the corresponding shear stress at this time. When the joint is instability and shear failure occur, the shear stress no longer decreases uniformly with time, but falls directly, which is the joint shear instability point under the stress path.

#### 3.3.2 Characteristics of displacement-time curve

**Fig 24:**
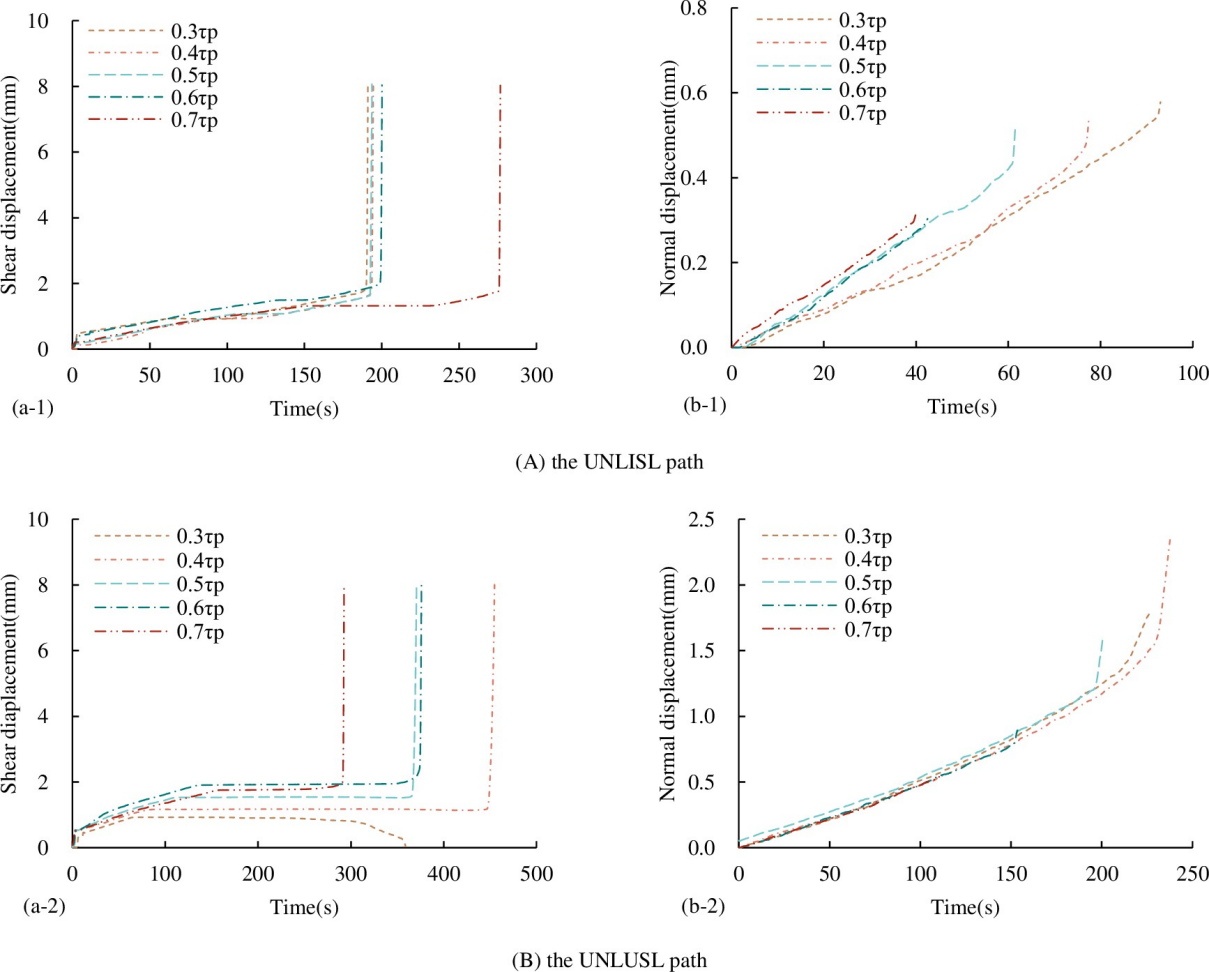
Displacement-time curves under different unloading stress paths (a) shear displacement-time curves (b) normal displacement-time curves.

The shear displacement-time curve of concrete joints under the UNLISL/UNLUSL path is shown in Fig 24 (excluding the first step of normal loading, only corresponding to the second step and the third step of the shear loading and unloading process).

The abrupt change of shear displacement and normal displacement in the displacement-time curve can be used to accurately determine the time point of shear instability failure. It can be seen from Fig 24(A) that the normal displacement-time curve under the UNLISL path is similar to that under the UNLCSL path, and the shear displacement-time curve is different. At the beginning of normal unloading, due to the loading in the shear direction, the shear displacement is no longer constant with the increase of time, and there is also a slight increase. Until a certain time, when the shear capacity of the joint is not enough to resist τ_1_, shear instability failure occurs, and the shear displacement increases instantaneously.

Fig 24(B) shows that under the UNLUSL path, the shear displacement-time curve and the normal displacement-time curve are similar to the UNLCSL path, which is no longer described here. However, since both the normal and shear stress are unloading processes, and σ_1_ is greater than τ_1_, according to the previous article, the normal stress at the beginning of unloading is 8 MPa, the shear stress is 2.13 MPa, the normal unloading rate is 2 MPa/min, and the shear unloading rate is 0.5 MPa/min. Even if the normal unloading rate is 4 times the shear unloading rate, τ_1_ is in the case of 0.3τ_p_, the stress difference at any time during the normal stress unloading to 0 MPa cannot make the joint destabilize. In the shear displacement-time curve, the shear displacement finally returns to the initial state of 0.

#### 3.3.3 Shear stress-shear displacement curve characteristics

**Fig 25:**
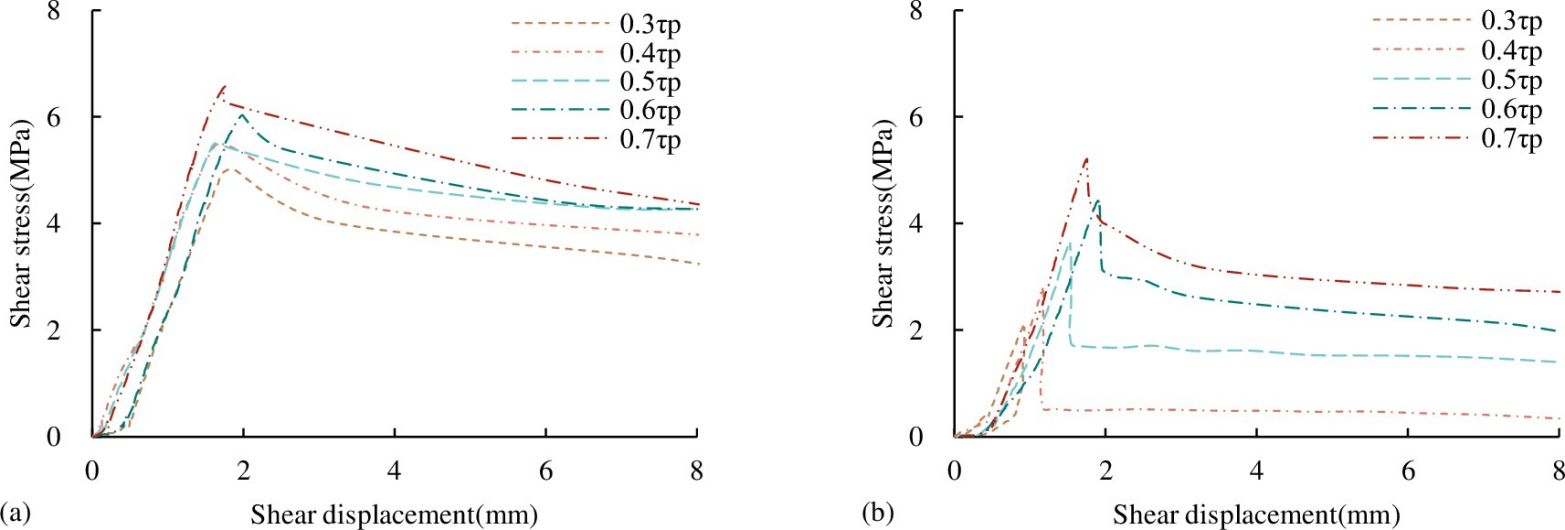
Shear stress-shear displacement curves under different unloading stress paths (a) the UNLISL path (b) the UNLUSL path.

Fig 25(A) is the shear stress-displacement curve obtained under the UNLISL path. Unlike the UNLCSL path, the peak point of the curve in Fig 25(A) is the instability point, and there is a significant shear stress drop after instability. Fig 25(B) is the shear stress-displacement curve obtained under the UNLUSL path. The peak point of the curve is only the starting point of the normal and shear unloading at the same time, and it is not the shear instability point of the concrete joint. The curve is difficult to judge the instability position, but it can be accurately judged by combining the stress time and displacement time curves. When the normal and shear unloading starts at the same time, the shear stress drops sharply, which is more obvious than the drop phenomenon under other stress paths, and there is no rebound stage.

#### 3.3.4 Analysis of shear mechanical parameters

According to the definition in 3.2.4, τ_1_, τ_*i*_ and σ_*i*_ under three unloading stress paths are obtained as shown in Table 7.

**Table 7:** Unloading direct shear test results of concrete joints under three stress paths.

*JRC*	Specimen number	*τ*_1_(MPa)	*τ*_*i*_ (MPa)	*σ*_*i*_ (MPa)	Δσ_n_	Stress path
11.06	ⅡC-0.3	2.23	2.07	1.80	6.20	UNLCSL
	ⅡC-0.4	3.04	2.93	2.78	5.22	
	ⅡC-0.5	3.62	3.47	3.94	4.06	
	ⅡC-0.6	4.37	4.21	4.55	3.45	
	ⅡC-0.7	4.96	4.71	5.23	2.77	
11.06	ⅢC-0.3	2.31	4.92	4.94	3.06	UNLISL
	ⅢC-0.4	3.08	5.43	5.39	2.61	
	ⅢC-0.5	3.86	5.51	5.97	2.03	
	ⅢC-0.6	4.63	6.03	6.29	1.71	
	ⅢC-0.7	5.4	6.25	6.44	1.56	
11.06	ⅣC-0.3	no instability occurred				UNLUSL
	ⅣC-0.4	3.08	0.52	0.09	7.91	
	ⅣC-0.5	3.86	1.65	1.35	6.65	
	ⅣC-0.6	4.63	2.84	2.87	5.13	
	ⅣC-0.7	5.4	3.81	4.27	3.73	

**Fig 26:**
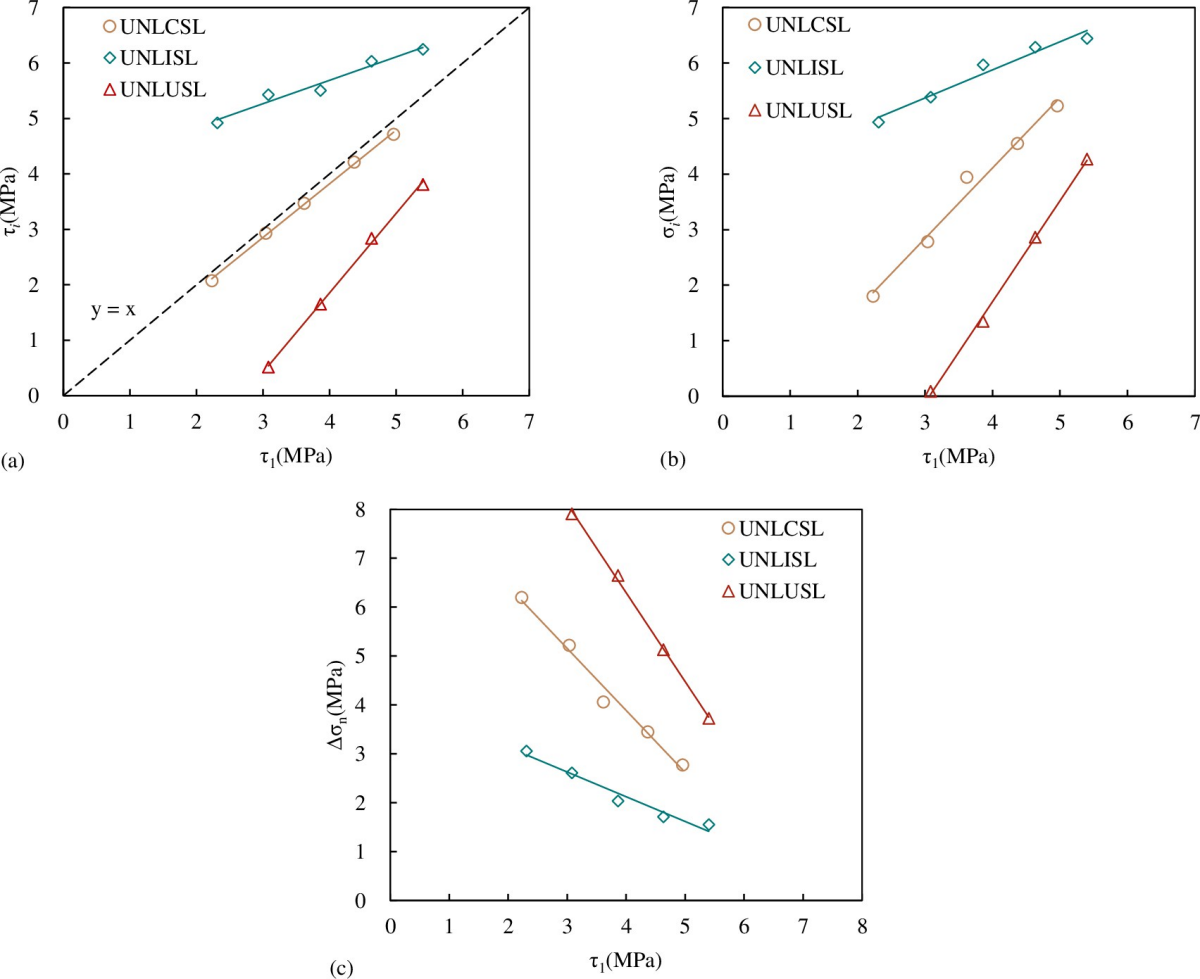
Relationship between σ_*i*_, τ_*i*_, Δσ_n_ and τ_1_ (a) the relationship between τ_*i*_ and τ_1_ (b) the relationship between σ_*i*_ and τ_1_ (c) the relationship between Δσ_n_ and τ_1_.

According to the results of Table 7, the relationship between σ_*i*_, τ_*i*_, Δσ_n_ and τ_1_ of the concrete joint specimen under three unloading stress paths is shown in Fig 26. When τ_1_is 0.3τ_p_, there is no joint instability failure in the specimen, so there are only four data points in the direct shear under the UNLUSL stress path in Fig 26. From Fig 26, it can be seen that under the same unloading stress path, σ_*i*_ and τ_*i*_ are approximately linear with τ_1_. The greater τ_1_, the greater σ_*i*_, and the larger the corresponding τ_*i*_. The Mohr-Coulomb criterion shows that in the loading test, the joint shear strength and the normal stress show a linear monotonic increasing trend, that is, the shear strength and the normal stress are one-to-one correspondence. Therefore, under the same τ_1_ when the shear stress increases (UNLISL group) τ_*i*_ increases. The corresponding σ_*i*_ is also be greater, and the normal stress Δσ_n_ is smaller. When the shear stress decreases (UNLUSL group), τ_*i*_ decreases the corresponding σ_*i*_ is also smaller, and Δσ_n_ also gets larger; when the shear stress is constant (UNLCSL group), the shear stress of the joint decreases slightly at the time of instability, the corresponding σ_*i*_ is in the middle, and Δσ_n_ is in the middle.

## 4. Comparative analysis

### 4.1 Strength characteristic

**Fig 27:**
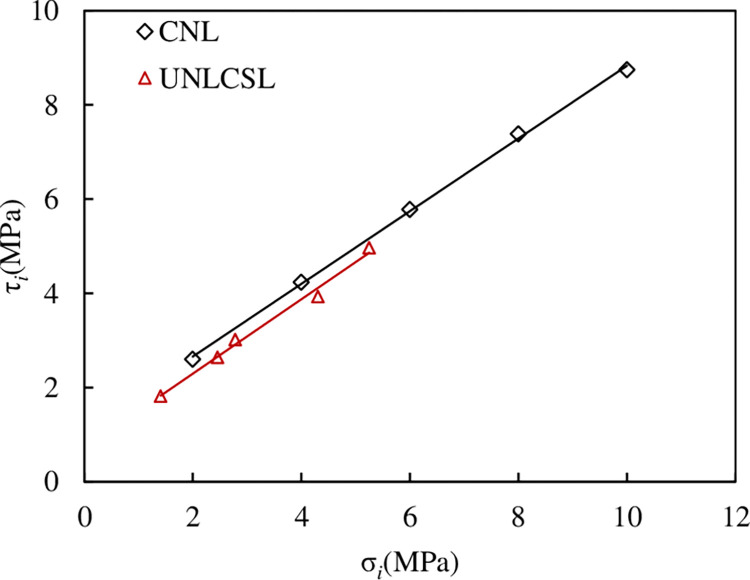
Comparison strength of the path of CNL and UNLCSL.

To compare the difference more intuitively in shear strength between the path of CNL and UNLCSL, we compare the test results obtained under the path CNL and UNLCSL for the same *JRC* (taking *JRC* = 11.06 as an example). The test results obtained under the two paths are compared in Fig 27.

It can be seen from Fig 27 that for the same *JRC* of the persistent joint when σ_*i*_ is same, τ_*i*_ under the UNLCSL path is significantly smaller than that under the CNL path. In the UNLCSL path, we will apply an τ_1_ in advance. After the joint is subjected to shear stress, a small part of the bulge will produce small damage to cause internal damage, so that when the joint is instability, τ_*i*_ of the joint is reduced. Another reason is that the normal unloading will lead to springback and dilatancy along the normal direction, and the resulting tensile stress will also lead to a decrease in the strength of the specimen.

Based on the strength law obtained by the CNL path, τ_t_ is obtained according to σ_*i*_, which is listed in Table 6. For the smooth joint (*JRC* = 0), τ_*i*_ obtained under the UNLCSL path is the same as the shear strength obtained under the CNL condition. With the increase of *JRC*, the deviation between τ_*i*_ and τ_t_ is increasing.

### 4.2 Characteristics of shear strength parameters

**Fig 28:**
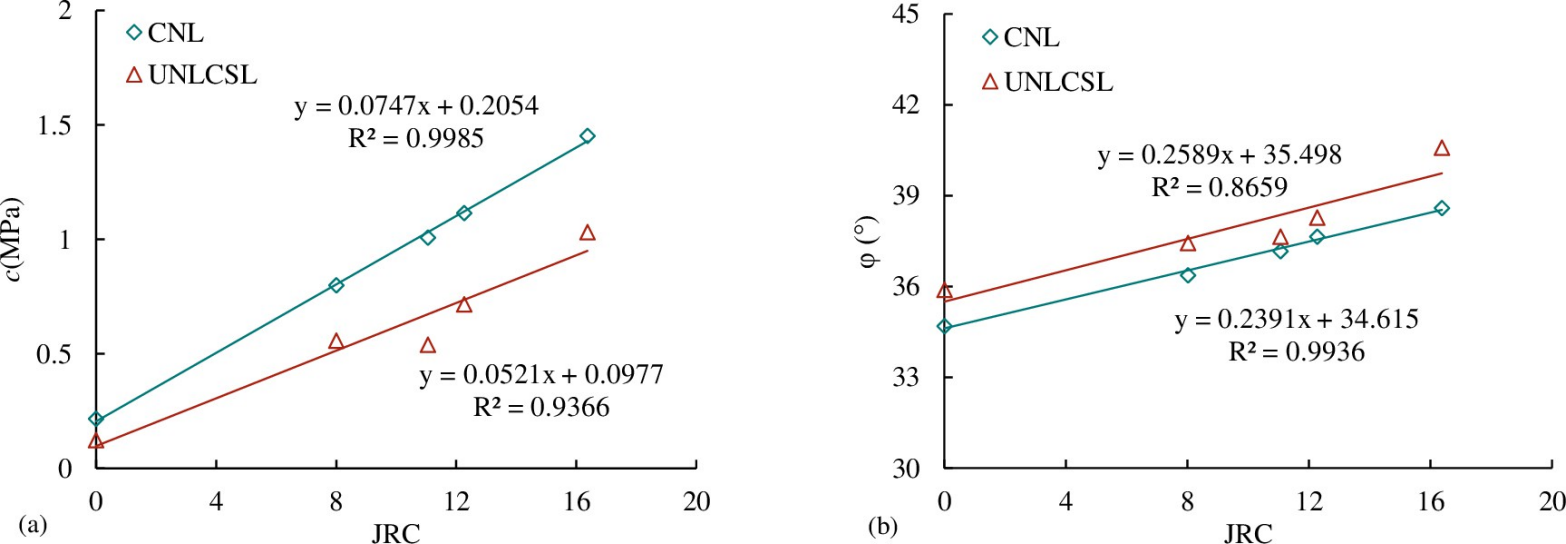
Comparison of shear strength parameters of direct shear tests under the path of CNL and UNLCSL (a) Relationship between joint cohesion *c* and *JRC* (b) Relationship between joint internal friction angle *φ* and *JRC*.

The relationship between the shear strength parameters and *JRC* under the path of CNL and UNLCSL respectively obtained. We compared and analyzed the shear strength parameters under the two paths, and obtained the relationship between the shear strength parameters and *JRC* under the two paths as shown in Fig 28.

Fig 28 shows that the comprehensive shear strength increases with the increase of *JRC*, whether it is normal stress or direct shear under normal unloading conditions. This is because the larger the *JRC* is, the more the protrusions of the joint are, the larger the contact area of the upper and lower joint surfaces is when the joint is sheared, and some large protrusions lead to the increase of the climbing amount when the joint is sheared, so the shear strength parameters increase with the increasing *JRC*. Under the same *JRC*, the cohesion under the UNLCSL path is less than the CNL path, and the internal friction angle is higher than that the CNL path. This is because in the unloading direct shear test, the joint will be affected by τ_1_ applied. Because the shear stress at this time is significantly lower than the bearing capacity of the joint, the joint will not immediately undergo shear instability failure. However, in the process of applying τ_1_, the specimen will have a climbing effect. Compared with the conventional direct shear test, the climbing effect of the unloading direct shear test is more obvious, resulting in a smaller area of convex contact between the upper and lower joint surfaces, resulting in the normal unloading.

### 4.3 Shear strength formula of concrete joint instability under unloading stress path

#### 4.3.1 Relationship between τ_*i*_ and σ_*i*_

**Fig 29:**
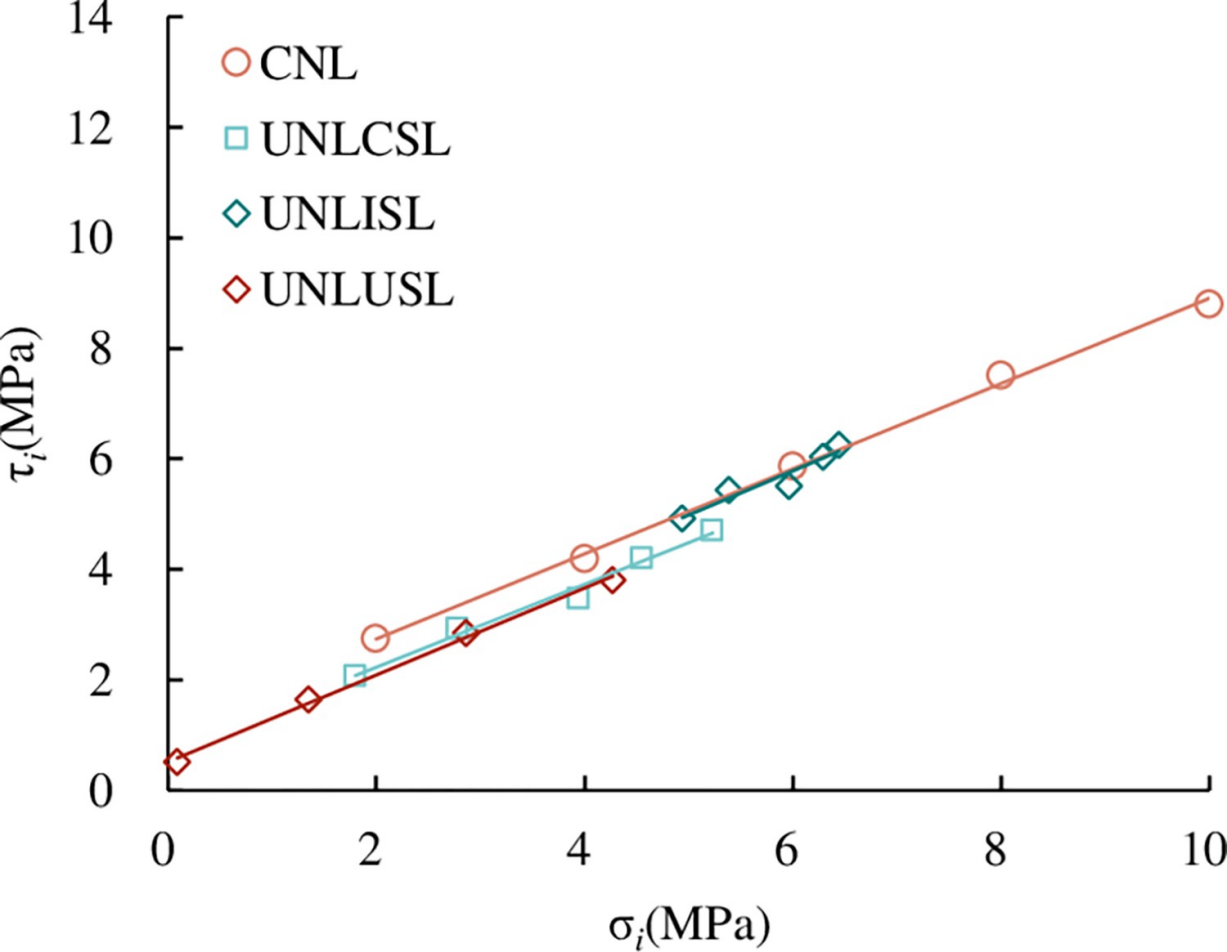
Relationship between τ_*i*_ and σ_*i*_.

The relationship between τ_*i*_ and σ_*i*_ is given in Fig 29. It can be seen from Fig 29 that the relationship between τ_*i*_ and σ_*i*_ can be expressed in the form of Mohr-Coulomb criterion. The formula is:

$$\tau_{i}=\sigma_{i}\;\tan\;\varphi +c$$ (7)

where *φ* is the internal friction angle of the joint, °; *c* is the joint cohesion, MPa.

It can be seen from Fig 29 that τ_*i*_ increases approximately linearly with the increase of σ_*i*_, and both of them also conform to the Mohr-Coulomb criterion. The fitting results of each curve are shown in Table 8. From the spatial relationship of the fitting line, under the same *JRC* and σ_*i*_, τ_*i*_ is the largest under the path of CNL and UNLISL, followed by the UNLCSL path, and τ_*i*_ under the UNLUSL path is the smallest. In general, it can be considered that under the same normal stress, τ_*i*_ obtained by the CNL path is greater than that under normal unloading conditions (which is consistent with the conclusions of Q.Z. Zhang [48] and M.L. Zhai [49]).

**Table 8:** Fig 29 in each curve fitting results.

Load path	Fitting result	*R* ^2^
CNL	*y* = 0.7707*x*+1.199	0.9981
UNLCSL	*y* = 0.751*x*+0.7293	0.9845
UNLISL	*y* = 0.7972*x*+0.9997	0.9146
UNLUSL	*y* = 0.7869*x*+0.5166	0.9968

#### 4.3.2 Relative error analysis of joint τ_*i*_

For concrete joints made of the same material with the same joint morphology, the loading and unloading stress path has a certain influence on the shear strength of joint instability. In engineering practice, the Mohr-Coulomb formula obtained under the CNL path is directly used to evaluate the stability of rock mass engineering without considering the loading and unloading stress path, which may overestimate the shear bearing capacity of joints and bring some risks to the evaluation of the stability of engineering rock mass.

To quantitatively analyze the error of the shear strength formula of joint instability under different unloading stress paths, this paper uses the average relative error to evaluate the applicability of the Mohr-Coulomb formula to predict the shear strength of joint instability under various control modes and unloading stress paths [23]. The average relative error expression is given as follows:

$$\delta =\frac{1}{n}\sum_{j=1}^{n}\left|\frac{\tau_{tj}-\tau_{ij}}{\tau_{tj}}\right|\times 100\%$$ (8)

where *δ* is the average value of relative error, %; *τ*_*tj*_ is the experimental value of the shear strength of the instability under each normal stress, MPa; *τ*_*ij*_ is τ_t_: based on the strength law obtained from the CNL path, the shear strength is obtained according to σ_*i*_, MPa; *n* is the number of shear tests for each group of specimens, and the number of tests for each group in this paper is 5 times. The average relative error of τ_*i*_ of the four groups of specimens is obtained as shown in Table 9.

**Table 9:** Average relative error of τ_*i*_.

Load path	CNL	UNLCSL	UNLISL	UNLUSL
average relative error *δ*/%	8.21	16.77	2.12	55.24

It can be seen from Table 9 that the average relative error of τ_*i*_ of the UNLUSL path is the largest, followed by the UNLCSL path, and the average relative error of the UNLISL path is the smallest. The average relative error of the joint τ_*i*_ of the UNLUSL path reached 55.24%, which will seriously lead to the overestimation of the joint τ_*i*_, thus bringing a higher risk to the evaluation of the stability of the engineering rock mass. Therefore, it is necessary to consider the influence of loading and unloading stress paths on the shear strength of joint instability when using the Mohr-Coulomb criterion to evaluate the stability of jointed rock mass.

#### 4.3.3 Modified shear strength formula of joint instability

From Fig 29 and Table 9, the slope of the fitting line between τ_*i*_ and the normal stress of the four groups of specimens is relatively close, and the difference in the intercept of the fitting line is more obvious. Therefore, it can be considered that the loading and unloading stress path has little effect on the internal friction angle of the joint specimen, and the decrease of τ_*i*_ mainly depends on the decrease of the joint cohesion. To further analyze the relationship between the reduction of joint τ_*i*_ and joint cohesion and internal friction angle, it is necessary to quantitatively analyze the joint cohesion and internal friction angle under different loading and unloading stress paths. Similarly, taking the test data under the CNL condition as the control group, the percentage reduction of the cohesion *c* and the internal friction angle *φ* of the specimen under the three unloading stress paths relative to the control group *R*_*c*_ and *R*_*φ*_ are obtained as shown in Table 10. Taking the internal friction angle as an example, the calculation formula of the reduction percentage *R*_*φ*_ is:

$$R_{\varphi }=\frac{\varphi_{i}-\varphi_{Α}}{\varphi_{A}}\times 100\%$$ (9)

where *R*_*φ*_ is the percentage of internal friction angle reduction, %; *φ*_A_ is the internal friction angle obtained by conventional direct shear test, °; *φ*_*i*_ is the internal friction angle of group i, °. When *R*_*φ*_ is greater than 0, it indicates that the internal friction angle increases relatively, and when *R*_*φ*_ is less than 0, it indicates that the internal friction angle decreases relatively.

**Table 10:** Percentage reduction of shear strength parameters.

Load path	UNLCSL	UNLISL	UNLUSL
angle of internal friction *R*_*φ*_/%	-1.9	2.5	1.5
force of cohesion *R*_*c*_/%	-39.2	-16.6	-56.9

It can be seen from Table 10 that under different unloading stress paths, compared with the CNL path, the cohesion of other groups is reduced. The smallest percentage of reduction is UNLISL test group 16.6%, and the largest is UNLUSL test group 56.9%, with an average of 37.6%. Under different unloading stress paths, the variation range of the internal friction angle of the specimen is small. From the above analysis, the change range of the internal friction angle of the specimen is within 3%, and the average decrease percentage of the cohesion of each group of specimens reach 37.6%. Therefore, the role of internal friction angle in the reduction of joint τ_*i*_ can be ignored, and the reduction of joint τ_*i*_ can be described only from the perspective of cohesion.

Keeping the internal friction angle unchanged, the correction coefficient k is introduced to reduce the cohesion, and the modified Mohr-Coulomb criterion formula is obtained as follows:

$$\tau =\sigma_{n}\;\tan\;\varphi +c_{i}$$ (10)

where *c*_*i*_ is the cohesive force, MPa, of the modified group *i* specimen, and its value is:

$$c_{i}=kc_{A}$$ (11)

where *k* is the cohesive force correction coefficient; *c*_A_ is the cohesion obtained by the conventional direct shear test under the joint morphology, MPa.

According to Formula (11), the correction coefficients of cohesion under three unloading stress paths are obtained, as shown in Table 11. The modified Mohr-Coulomb criterion correction formula of joint shear instability under three unloading stress paths can be obtained by substituting the cohesion of each group of specimens into Formula (10). Based on the above Mohr-Coulomb criterion correction formula, the average relative error of joint τ_*i*_ under three unloading stress paths can be obtained, as shown in Table 12.

**Table 11:** Cohesion correction coefficient.

Load path	UNLCSL	UNLISL	UNLUSL
cohesion correction coefficient	0.61	0.83	0.43

**Table 12:** Average relative error of τ_*i*_.

Load path	CNL	UNLCSL	UNLISL	UNLUSL
average relative error δ / %	8.21	2.2	3.5	5.9

It can be seen from Table 11 that after introducing the cohesive force correction coefficient *k*, the average relative error of τ_*i*_ under each unloading stress path calculated by Formula (10) is smaller than that of Formula (7). The maximum value of the average relative error after correction is only 3.5%, which is significantly improved compared with the maximum value of the average relative error before correction of 56.9%. The introduction of the cohesion correction coefficient *k* to modify the Mohr-Coulomb criterion can provide a new idea for the reasonable estimation of the shear strength of the joint specimen under the corresponding unloading stress path. Specifically, in the stability evaluation of jointed rock mass such as slope engineering and underground caverns, the unloading stress path borne by the joint can be confirmed first, and then the cohesion c in the Mohr-Coulomb criterion applicable to the joint can be reduced and corrected, and the Mohr-Coulomb correction formula suitable for the joint can be obtained.

## 5. Conclusion

(1) The peak shear strength, cohesion, internal friction angle, pre-peak shear stiffness and residual shear strength of concrete joints under CNL path increases with the increasing JRC and normal stress. With the increase of normal stress, the failure mode of the joint surface transits from wear failure to shear-off failure.

(2) Under the UNLCSL path, under the same initial shear stress τ_1_, instability normal stress σ_*i*_ decreases with the increasing *JRC*, and normal stress unloading amount Δσ_n_ increases with the increasing *JRC*. Under the same *JRC*, σ_*i*_ increases with the increase of τ_1_, and Δσ_n_ decreases with the increasing τ_1_. Under the same *JRC* and σ_*i*_, τ_*i*_ is significantly smaller under the UNLCSL path than the CNL path. Under the same *JRC*, the cohesion under the UNLCSL path is less than the CNL path, and the internal friction angle is higher than that the CNL path.

(3) Under the same *JRC* and σ_*i*_, τ_*i*_ is the largest under the CNL path and UNLISL, followed by the UNLCSL path, and τ_*i*_ under the UNLUSL path is the smallest. Compared with the CNL path, the variation range of the specimen internal friction angle is within 3% while the average decrease percentage of the specimen cohesion reaches 37.6% under the UNLCSL path, UNLISL, and UNLUSL. Therefore, it can be inferred that the decrease in cohesion caused by normal unloading is the main reason for the decrease in joint instability shear strength. After introducing the correction coefficient *k* of cohesion to modify the Mohr-Coulomb criterion, the maximum average relative error after correction is only 3.5%, which is significantly improved compared with the maximum average relative error of 56.9% before correction.

The research conclusions can provide some reference for the accurate estimation of shear bearing capacity of rock mass joints under different unloading stress paths, which is of great significance to the stability evaluation and disaster prevention of rock mass engineering.
